# Tailoring Intermediate Adsorption Through *d*‐band Mismatch Strategy of Heterojunction to Achieve Industrial Overall Water Splitting

**DOI:** 10.1002/advs.202521184

**Published:** 2026-01-12

**Authors:** Jia Liu, Jiawen Sun, Yi‐Ru Hao, Yaqin Chen, Chunhao Li, Le‐Le Ma, Jing Sun, Zhonglong Zhao, Jiangwei Zhang, Hui Xue, Qin Wang

**Affiliations:** ^1^ College of Chemistry and Chemical Engineering Inner Mongolia University Hohhot China; ^2^ College of Physical Science and Technology Inner Mongolia University Hohhot China; ^3^ College of Energy Materials and Chemistry Inner Mongolia Key Laboratory of Low Carbon Catalysis Inner Mongolia University Hohhot China

**Keywords:** ampere‐level current density, anion exchange membrane water electrolyzer, *d*‐band mismatch, heterojunction engineering, intermediate adsorption

## Abstract

Adjusting the *d*‐band center of catalysts through heterojunction construction represents an effective approach for enhancing the catalytic activity. Nevertheless, the precise modulation pathways of *d*‐band centers still require systematic elucidation. In this work, a Ni_3_Mo/Ni_3_N junction is constructed to investigate *d*‐band engineering, and a *d*‐band mismatch mechanism has been proposed for the first time to elucidate the synergistic effect between Ni_3_Mo and Ni_3_N for improved HER and OER. Specifically, the dissociation of H_2_O can be achieved on the Ni_3_Mo surface while the adjacent Ni_3_N sites catalyze the subsequent evolution reactions. Remarkably, the Ni_3_Mo–Ni_3_N/NF achieves ultra‐low overpotentials of 15 mV (HER) and 155 mV (OER) at 10 mA cm^−2^, and just 228 mV (HER) and 459 mV (OER) at 1 A cm^−2^. Most strikingly, the HER performance of Ni_3_Mo–Ni_3_N/NF is superior to that of the Pt/C catalyst across all current densities, marking it as a standout among the NiMo‐based catalysts documented so far. Additionally, the Ni_3_Mo–Ni_3_N/NF demonstrates outstanding performance in an anion exchange membrane water electrolyzer (AEMWE), delivering the current density of 4 A cm^−2^ at a mere 2.12 V while maintaining stable operation for 1000 h at 1 A cm^−2^, showing great potential for practical applications.

## Graphical abstract

This paper demonstrates the fabrication of a Ni_3_Mo/Ni_3_N junction to investigate *d*‐band engineering and elucidate the specific roles of Ni_3_Mo and Ni_3_N active sites in improving catalytic activity. Through integrated spectroscopic characterization and first‐principles modeling, for the first time, a *d*‐band mismatch mechanism has been proposed to elucidate the synergistic effect between Ni_3_Mo and Ni_3_N for improved HER and OER.

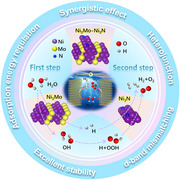

## 1 Introduction

Hydrogen energy has emerged as a highly compelling sustainable energy option, drawing considerable attention for its role as a zero‐emission alternative to conventional energy sources [1, 2, 3, 4, 5]. Water electrolysis, a high‐efficiency and pollution‐free hydrogen production technology, is widely recognized as a promising method for generating high‐purity hydrogen [6, 7, 8, 9]. However, the slow reaction kinetics of both the hydrogen evolution reaction (HER) and oxygen evolution reaction (OER) in water electrolysis processes pose a notable barrier to its large‐scale industrial implementation [10, 11, 12, 13, 14]. While precious metal‐based catalysts such as Pt/C and RuO_2_ display impressive catalytic activity, their scarce natural abundance and prohibitive cost remain major obstacles to large‐scale commercial adoption [15, 16, 17, 18, 19]. Hence, developing efficient and durable catalysts for water electrolysis is critically important.

The NiMo alloys demonstrate exceptional performance in water electrolysis due to the distinctive electronic architecture and abundant active sites. The water dissociation ability of pure Ni is moderate, while the Mo atom exhibits superior H adsorption ability [20]. Consequently, the enhanced HER performance of the NiMo alloy can be attributed to its comparable surface electronic state with the commercial Pt/C catalyst [21]. However, the catalytic performance of a single NiMo alloy remains suboptimal, prompting researchers to employ the strategy of combining two or more components to construct a heterogeneous structure, which has been demonstrated as an effective approach for enhancing catalytic activity [22, 23, 24]. According to the Langmuir‐Hinshelwood mechanism, in heterogeneous catalysis, the reactants initially adsorb onto the catalyst's surface and subsequently diffuse towards the active site, where they undergo a reaction to yield adsorption products prior to desorption [25]. The key determinant of the catalyst's activity is the adsorption strength of the intermediate products on the catalyst surface [26]. The construction of a heterogeneous interface is advantageous for fine‐tuning the electronic structure and metal *d*‐band centers, thereby directly impacting the adsorption free energy of intermediates [27, 28]. In the catalyst, activity can be assessed by the position of the *d*‐band center and its shift magnitude. The adsorption strength of HER and OER intermediates can be effectively modulated by precisely adjusting the *d*‐band centers, thereby facilitating an accelerated reaction rate for overall water splitting (OWS) [26]. However, specifying the distinct function of each component in most heterojunctions poses a challenge, and the effects of synergistic regulation between components on the *d*‐band center of metals remain unclear.

Herein, a Ni_3_Mo–Ni_3_N/NF heterojunction has been successfully fabricated to study the *d*‐band engineering, which exhibits remarkable activities in both HER and OER. Specifically, the overpotential of HER and OER on the Ni_3_Mo–Ni_3_N/NF is only 15 and 155 mV at 10 mA cm^−2^, respectively. Additionally, the catalyst also demonstrates outstanding bi‐functional activity by achieving low overpotentials in HER (228 mV) and OER (459 mV) at 1 A cm^−2^. It is noteworthy that the Ni_3_Mo–Ni_3_N/NF exhibits superior HER performance at all current densities compared to the Pt/C catalyst, marking it as a standout among the NiMo‐based catalytic materials documented so far. The Ni_3_Mo–Ni_3_N/NF also manifests exceptional stability, as evidenced by its ability to maintain HER and OER activity at 10 mA cm^−2^ for over 1000 h. The outstanding performance of Ni_3_Mo–Ni_3_N/NF in both HER and OER processes enables them to exhibit extremely high activity and stability during the OWS process. In 1 M KOH electrolyte, the catalyst achieves 10 and 1000 mA cm^−2^ at voltages as low as 1.39 and 1.79 V, respectively, while maintaining virtually unchanged properties in simulated seawater solutions. Additionally, 1000 h stability tests conducted at 10 and 1000 mA cm^−2^ confirm the exceptional stability of the Ni_3_Mo–Ni_3_N/NF electrolyzer. Furthermore, the Ni_3_Mo–Ni_3_N/NF demonstrates outstanding performance in an anion exchange membrane water electrolyzer (AEMWE), reaching 4 A cm^−2^ at a low voltage of 2.12 V while maintaining stable operation for 1000 h at 1 A cm^−2^ under room temperature, showing great potential for applications. Density functional theory (DFT) calculations demonstrate that the excellent activity of Ni_3_Mo–Ni_3_N/NF is primarily attributed to the synergistic optimization of its Ni_3_Mo and Ni_3_N components, which enables rapid dissociation of H_2_O molecules into H* and OH* intermediates on the Ni_3_Mo site, followed by further reactions on the Ni_3_N site to produce H_2_ and O_2_. Meanwhile, this work proposes the *d*‐band mismatch mechanism for the first time. Guided by this mechanism, we further reveal that heterojunction construction reduces the *d*‐band centers of the five Ni *d*‐orbitals in the Ni_3_N sites of Ni_3_Mo–Ni_3_N/NF relative to those in the Ni sites of Ni_3_Mo, thereby elucidating the weakened adsorption of hydrogen and oxygen intermediates. The study realizes the mismatch of metal *d*‐band centers by constructing heterogeneous interfaces, thereby effectively modulating the adsorption energy of intermediates, which offers insights into the electronic structure and *d*‐band regulation of electrocatalysts.

## 2 Results and Discussion

**FIGURE 1:**
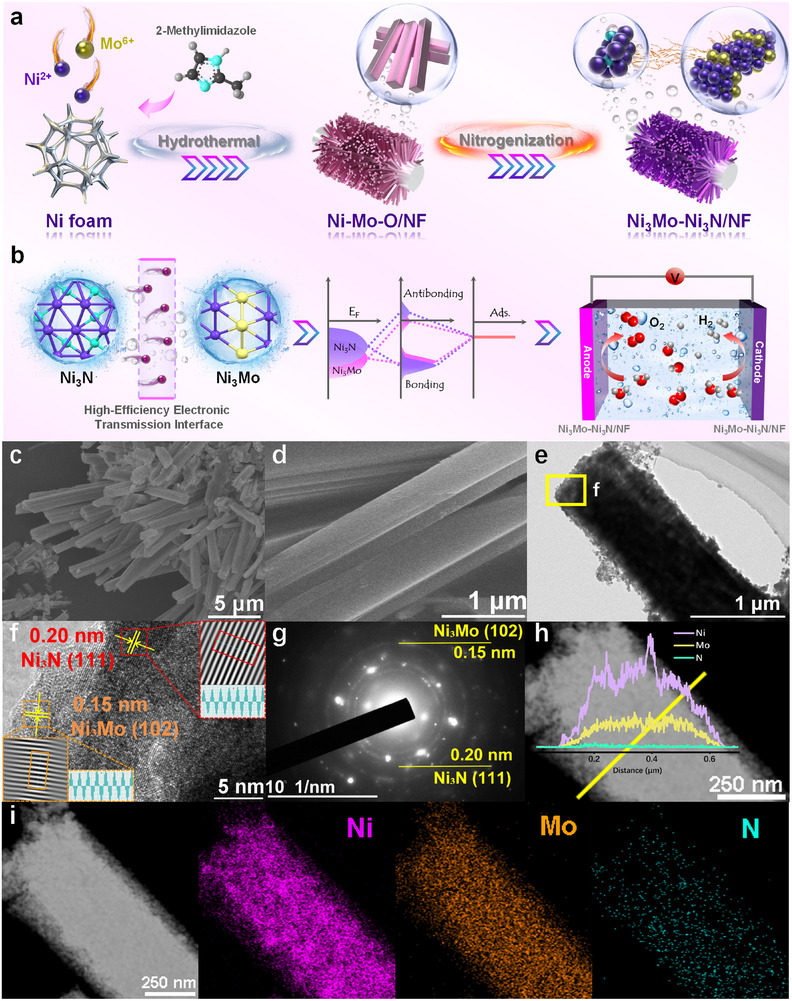
(a,b) Synthesis schematic, (c,d) SEM images, (e) TEM image, (f) HR–TEM image, (g) SAED, (h–i) EDS line sweep and elemental mapping of Ni_3_Mo–Ni_3_N/NF.

The heterostructured Ni_3_Mo–Ni_3_N/NF was fabricated via sequential hydrothermal growth and nitridation, as illustrated in Figure 1a. Firstly, Ni oxide and Mo oxide precursors were synthesized on a nickel foam substrate via the hydrothermal method. Subsequently, the utilization of melamine in a tubular furnace for nitriding leads to the formation of a heterojunction comprising Ni_3_Mo and Ni_3_N. Such a heterostructure with mismatched *d*‐bands can effectively regulate the adsorption of reaction intermediates, thereby significantly influencing the catalytic activity (Figure 1b). Phase characterization of the catalyst was performed via X‐ray powder diffraction (XRD), with the corresponding results presented in Figure S1. The presence of diffraction peaks indicates the inclusion of Ni_3_Mo (JCPDS Card No. 50–1094) and Ni_3_N (JCPDS Card No. 10–0280) in the catalyst. The diffraction peaks located at 42.2°, 45.1°, and 48.8° align with the (002), (101), and (102) crystal planes of Ni_3_Mo, while those at 44.5°, 70.6°, and 78.4° match the (111), (300), and (113) faces of Ni_3_N. The surface morphology and structure of the catalysts were observed through scanning electron microscopy (SEM) and transmission electron microscopy (TEM). The nanorod‐like structure of the synthesized catalyst is visible in Figure 1c–e and Figures S2–S4. The high‐resolution TEM (HR–TEM) image (Figure 1f) and selected area electron diffraction (SAED) image (Figure 1g) reveal that the crystal plane spacing of Ni_3_Mo is determined to be 0.15 nm, corresponding to the (102) crystal phase, while for Ni_3_N, it is measured as 0.20 nm, corresponding to the (111) crystal phase. Furthermore, the line sweep mapping in Figure 1h and energy dispersive spectroscopy (EDS) mapping images in Figure 1i exhibit a homogeneous distribution of Ni, Mo, and N elements.

**FIGURE 2:**
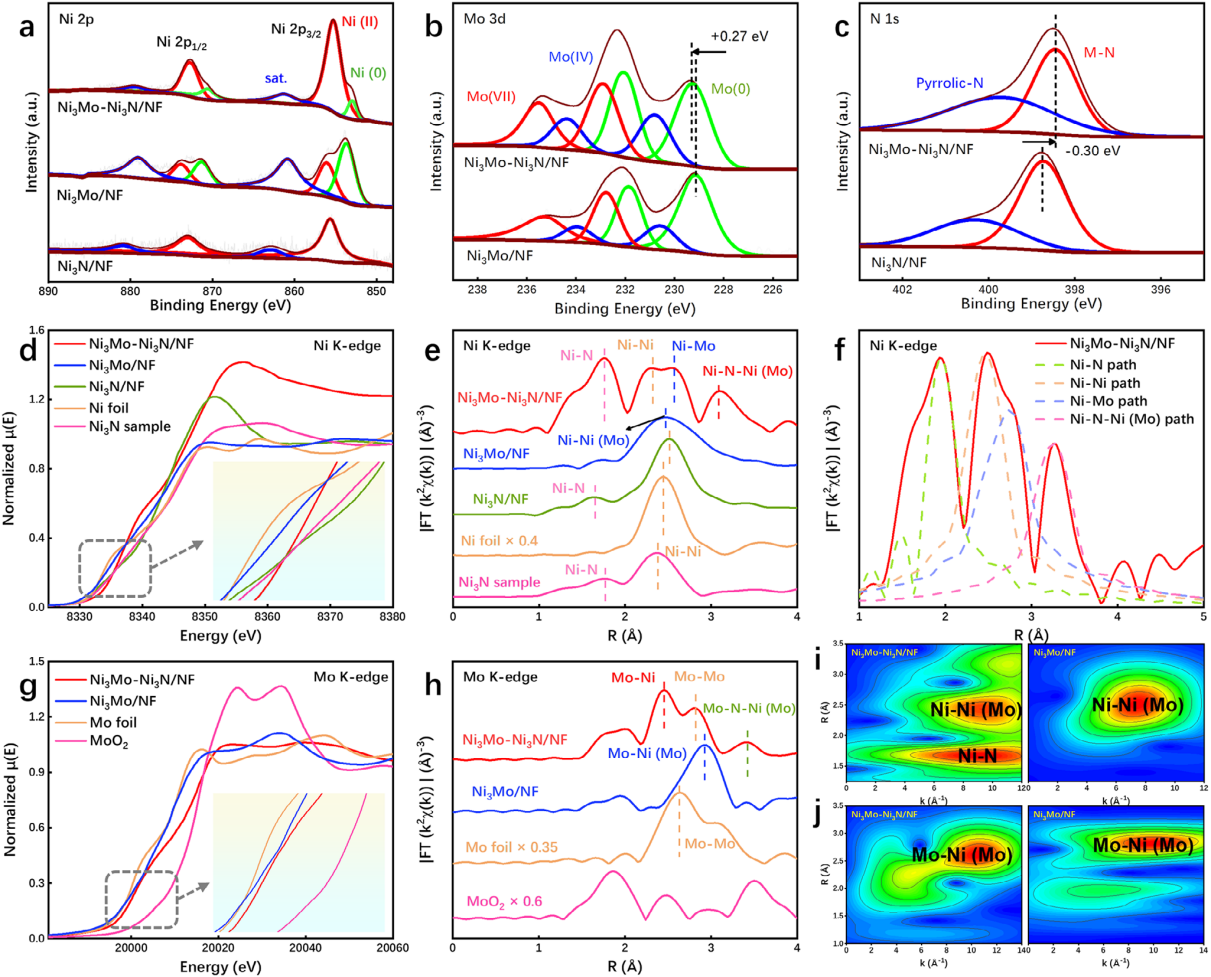
XPS spectra of the Ni_3_Mo–Ni_3_N/NF, Ni_3_Mo/NF, and Ni_3_N/NF catalysts for (a) Ni 2*p*, (b) Mo 3*d*, and (c) N 1*s*. (d) XANES spectrum and (e) Fourier‐transformed EXAFS k^2^𝜒(k) data of Ni K‐edges, (f) Scattering path of Ni_3_Mo–Ni_3_N/NF Ni K‐edge. (g) XANES spectrum and (h) Fourier‐transformed EXAFS k^2^𝜒(k) data of Mo K‐edges. (i) WT‐EXAFS plots of Ni_3_Mo–Ni_3_N/NF and Ni_3_Mo/NF in Ni K‐edges. (j) WT‐EXAFS plots of Ni_3_Mo–Ni_3_N/NF and Ni_3_Mo/NF in Mo K‐edge.

The X‐ray photoelectron spectroscopy (XPS) analysis confirms the surface composition of Ni_3_Mo–Ni_3_N/NF, identifying Ni, Mo, N, and C elements (Figure S5). In Figure 2a, the XPS spectra of Ni 2*p* show two distinct spin–orbit double peaks of Ni 2*p*_3/2_ and Ni 2*p*_1/2_. The peaks at 853.1 (Ni 2*p*_3/2_) and 870.5 eV (Ni 2*p*_1/2_) are characteristic of metallic Ni (Ni^0^) [29, 30]. Likewise, the peaks observed at 855.3 (Ni 2*p*_3/2_) and 872.7 eV (Ni 2*p*_1/2_) are indicative of Ni^2+^ species [31]. The Mo 3*d* XPS spectra exhibit the presence of Mo^0^, Mo^4+^, and Mo^6+^, respectively (Figure 2b) [32, 33]. The observed Ni^0^ and Mo^0^ offer additional support for the formation of alloys [34]. The characteristic peak of Mo^0^ at 229.2 eV exhibits a positive shift of 0.27 eV subsequent to the formation of the heterojunction, indicating an electron loss at the Mo site and a reduction in the density of the surrounding electron cloud [21, 35]. In the N 1*s* XPS spectra, two distinct characteristic peaks appearing at 398.7 and 400.2 eV align with metal‐bonded N and pyrrole N, respectively [26, 36]. Following the formation of a heterojunction, there is a negative shift of 0.23 eV in the characteristic peak associated with metal N, indicating electron acquisition by the N site due to heterojunction formation and an increase in the density of the surrounding electron cloud (Figure 2c) [9, 26]. The results suggest that there is a continuous electron transfer occurring at the interface of the Ni_3_Mo–Ni_3_N/NF, which facilitates effective regulation of the electronic structure and enhances catalytic activity [37]. Subsequently, in Figure S6, the *d*‐band center of the Ni_3_Mo–Ni_3_N/NF was determined to be −5.24 eV using UV photoelectron spectroscopy (UPS), which was found to be smaller than that of Ni_3_Mo (−4.80 eV). The *d*‐band center theory reveals the coupling relationship between the oxidation state of the adsorbate and the *d*‐band center of the catalyst. The proximity of the *d*‐band center to the Fermi level leads to an increased availability of empty antibonding states, thereby enhancing the adsorption strength of intermediates [38]. The formation of a heterojunction leads to a decrease in the *d*‐band center, thereby attenuating the binding energy between the catalyst and the adsorbate, thus facilitating intermediate desorption [39, 40, 41, 42].

To decipher the local coordination environment of the obtained catalysts, we further conducted X‐ray absorption spectroscopy (XAS) analyses. In Figure 2d, the Ni K‐edge X‐ray absorption near‐edge structure (XANES) spectra indicate that the adsorption edge of Ni_3_Mo and Ni_3_N exhibits distinct edge absorption characteristics similar to those of Ni foil and standard Ni_3_N, respectively. Meanwhile, Ni_3_Mo–Ni_3_N/NF is located between Ni foil and the standard Ni_3_N sample, suggesting the average valence states of Ni in Ni_3_Mo–Ni_3_N/NF are between 0 and +3. Moreover, Ni K‐edge extended X‐ray absorption fine structure (EXAFS) spectra were used to elucidate the coordination structure of Ni, with the corresponding spectra presented in Figure 2e. The presence of a peak at 1.89 Å in Ni_3_Mo–Ni_3_N/NF and Ni_3_N/NF is consistent with the characteristic peak observed for the standard Ni_3_N sample, which matches the coordination of Ni─N in the first shell. As shown in Figure 2f and Figure S7 and Table S1, the presence of peaks at 2.47, 2.73, and 3.26 Å in Ni_3_Mo–Ni_3_N/NF corresponds to the Ni─Ni coordination in the second shell, the Ni─Mo coordination in the third shell, and the Ni─N─Ni (Mo) coordination in the fourth shell, respectively. The XANES spectrum of the Mo K‐edge reveals that the absorption edge of Ni_3_Mo–Ni_3_N/NF is located close to the Mo foil, demonstrating that the oxidation state of Mo is close to 0 (Figure 2g). The local coordination environment of Mo in Ni_3_Mo–Ni_3_N/NF was further studied using extended EXAFS. As depicted in Figure 2h and Figure S8 and Table S2, the peaks observed at 2.65, 2.81, and 3.42 Å can be attributed to the Mo─Ni bond, Mo─Mo bond, and Mo─N─Ni (Mo) bond, respectively. Subsequently, the coordination environment of Ni and Mo in different samples is further analyzed using the continuous wavelet transform (WT). The scattering path signal in Figure 2i,j and Figure S9 for Ni_3_Mo–Ni_3_N/NF, Ni_3_Mo/NF, and Ni_3_Mo/NF aligns with the findings from previous analyses.

**FIGURE 3:**
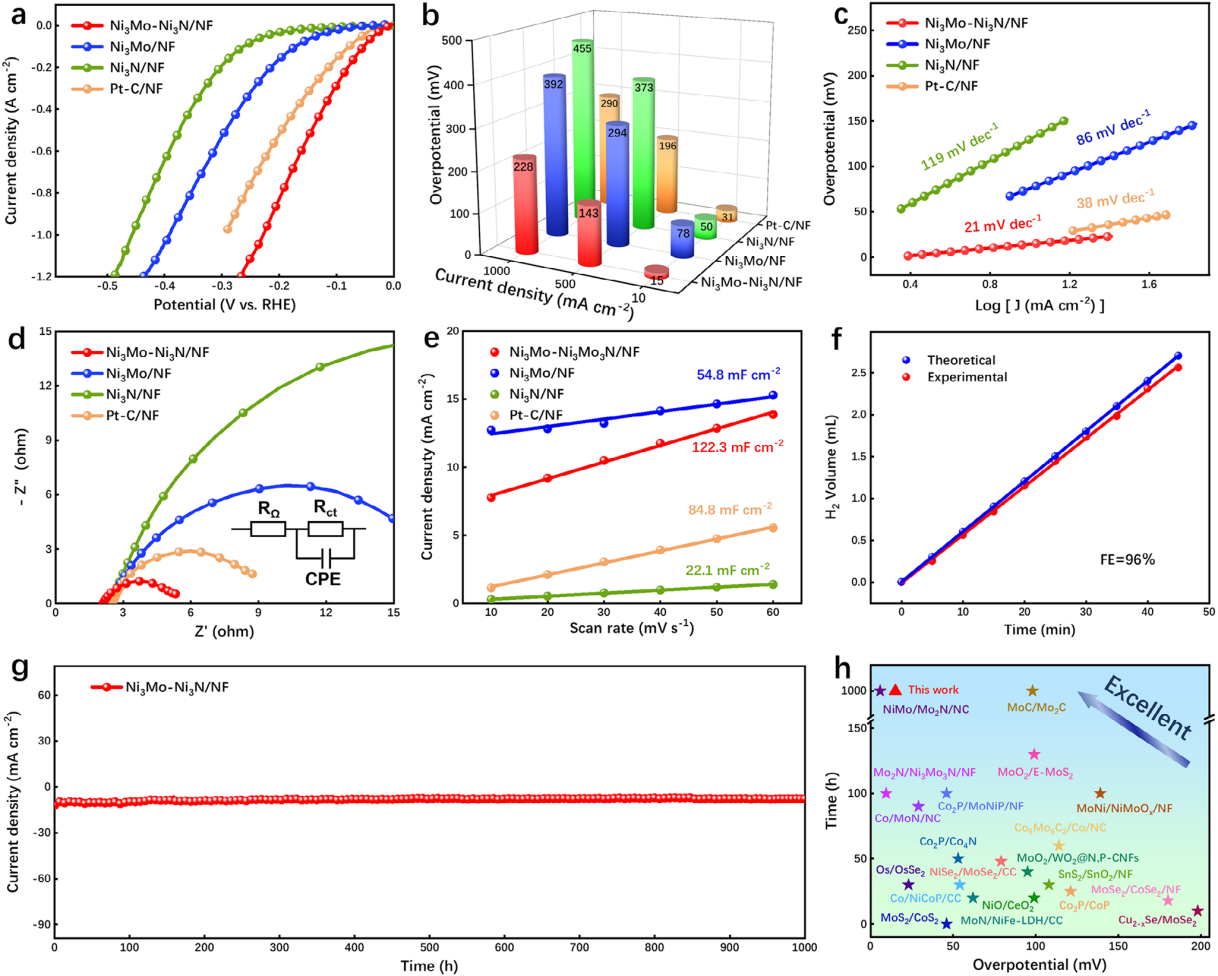
HER electrochemical characterization in 1 M KOH electrolyte, (a) LSV curves, (b) HER overpotentials at different current densities, (c) Tafel slopes, (d) Nyquist plots, and (e) C_dl_ values of Ni_3_Mo–Ni_3_N/NF, Ni_3_Mo/NF, Ni_3_N/NF, and Pt‐C/NF. (f) Faraday efficiency of Ni_3_Mo–Ni_3_N/NF. (g) *I–t* stability test of the Ni_3_Mo–Ni_3_N/NF during 1000 h at 10 mA cm^−2^. (h) Comparison of recently reported catalysts for HER.

The HER performance of the obtained samples was assessed in 1 M KOH. As depicted in Figure 3a,b, the Ni_3_Mo–Ni_3_N/NF demonstrates a significantly reduced overpotential of merely 15 mV at 10 mA cm^−2^ compared to the comparative samples Ni_3_Mo/NF (78 mV), Ni_3_N/NF (50 mV), and Pt‐C/NF (31 mV). Additionally, the Ni_3_Mo–Ni_3_N/NF heterostructure exhibits exceptional HER performance at ampere‐level current density, achieving 1 A cm^−2^ with a low overpotential of merely 228 mV. Moreover, the Tafel slope of 21 mV dec^−1^ is lower than those of Ni_3_Mo/NF (86 mV dec^−1^), Ni_3_N/NF (119 mV dec^−1^), and Pt‐C/NF (38 mV dec^−1^), as shown in Figure 3c. Electrochemical impedance spectroscopy (EIS) was used to assess the catalyst's charge transport capability. In Figure 3d and Figure S12, and Tables S3–S6, the Ni_3_Mo–Ni_3_N/NF catalyst after fitting displays the smallest charge transfer resistance (R_ct_) (3.204 Ω), suggesting that heterojunction formation lowers the electron transfer energy barrier between the catalyst and electrolyte, thus accelerating the reaction kinetics. Additionally, electrochemical double layer capacitance (C_dl_) is determined through CV measurements conducted at various sweep rates (Figure S13), enabling the investigation of the impact of electrochemical surface areas (ECSA) on HER (Figure S14; Tables S7 and S8). The Ni_3_Mo–Ni_3_N/NF exhibits a significantly higher C_dl_ value of 122.3 mF cm^−2^ (Figure 3e), surpassing that of Ni_3_Mo/NF (54.8 mF cm^−2^), Ni_3_N/NF (22.1 mF cm^−2^), and Pt‐C/NF (84.8 mF cm^−2^), which suggests that the heterojunction possesses the largest ECSA. The Faraday efficiency test was conducted using the drainage gas collection method, and the obtained results demonstrate that the Ni_3_Mo–Ni_3_N/NF exhibits an HER Faraday efficiency of 96% (Figure 3f). The electrochemical stability of the Ni_3_Mo–Ni_3_N/NF catalyst was assessed through a chronoamperometry (*i–t*) test. The results depicted in Figure 3g demonstrate that the current density remains unchanged even after 1000 h of reaction at 10 mA cm^−2^, indicating its exceptional stability. As illustrated in Figure 3h and Table S9, in comparison to the recently reported Mo‐ and Ni‐based electrocatalysts, the Ni_3_Mo–Ni_3_N/NF exhibits remarkable HER activity and stability.

**FIGURE 4:**
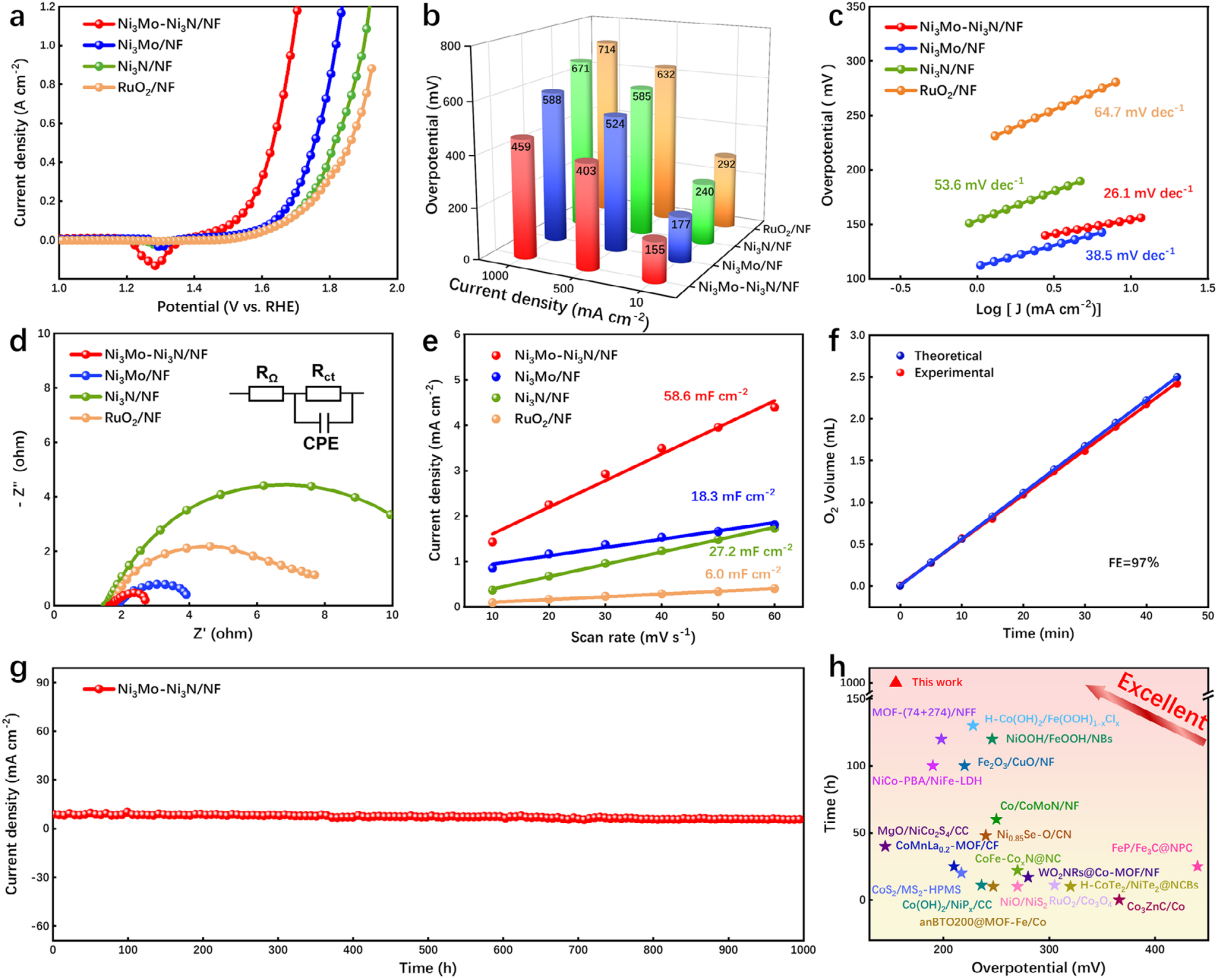
OER electrochemical characterization in 1 M KOH electrolyte, (a) LSV curves, (b) OER overpotentials at different current densities, (c) Tafel slopes, (d) Nyquist plots, and (e) C_dl_ values of Ni_3_Mo–Ni_3_N/NF, Ni_3_Mo/NF, Ni_3_N/NF, and RuO_2_/NF. (f) Faraday efficiency of Ni_3_Mo–Ni_3_N/NF. (g) *I–t* stability test of the Ni_3_Mo–Ni_3_N/NF during 1000 h at 10 mA cm^−2^. (h) Comparison of recently reported catalysts for OER.

The OER catalytic activity of the different catalysts was also tested in a 1 M KOH electrolyte. As depicted in Figure 4a,b, the Ni_3_MoNi_3_N/NF exhibits remarkable OER activity, characterized by a low overpotential of 155 mV at 10 mA cm^−2^, outperforming Ni_3_Mo/NF (177 mV), Ni_3_N/NF (240 mV), and RuO_2_/NF (292 mV). Meanwhile, even when the current density is increased to 1 A cm^−2^, the overpotential of the Ni_3_Mo–Ni_3_N/NF catalyst remains as low as 459 mV. Moreover, the remarkably low Tafel slope exhibited by 26.1 mV dec^−1^ for Ni_3_Mo–Ni_3_N/NF indicates its superior OER reaction kinetics in Figure 4c. The EIS and fitting results presented in Figure 4d and Figure S15 and Tables S10–S13 demonstrate that the R_ct_ (1.143 Ω) value of Ni_3_Mo–Ni_3_N/NF is the lowest, indicating superior electron transport capability during the OER process. The largest C_dl_ of 58.6 mF cm^−2^ (Figure 4e; Figure S16) and ECSA (Figure S17; Table S14) for Ni_3_Mo–Ni_3_N/NF further substantiates the abundant active sites created by the formation of a heterojunction. In Figure 4f, the Ni_3_Mo–Ni_3_N/NF catalyst shows an OER Faraday efficiency of 97%, attesting to its exceptional performance in oxygen evolution. The chronoamperometry test result demonstrates that the Ni_3_Mo–Ni_3_N/NF exhibits excellent stability (Figure 4g), maintaining its performance at 10 mA cm^−2^ for 1000 h. The exceptional OER activity and stability of the Ni_3_Mo–Ni_3_N/NF are further elucidated compared to the majority of previously reported non‐noble metal catalysts (Figure 4h; Table S15).

**FIGURE 5:**
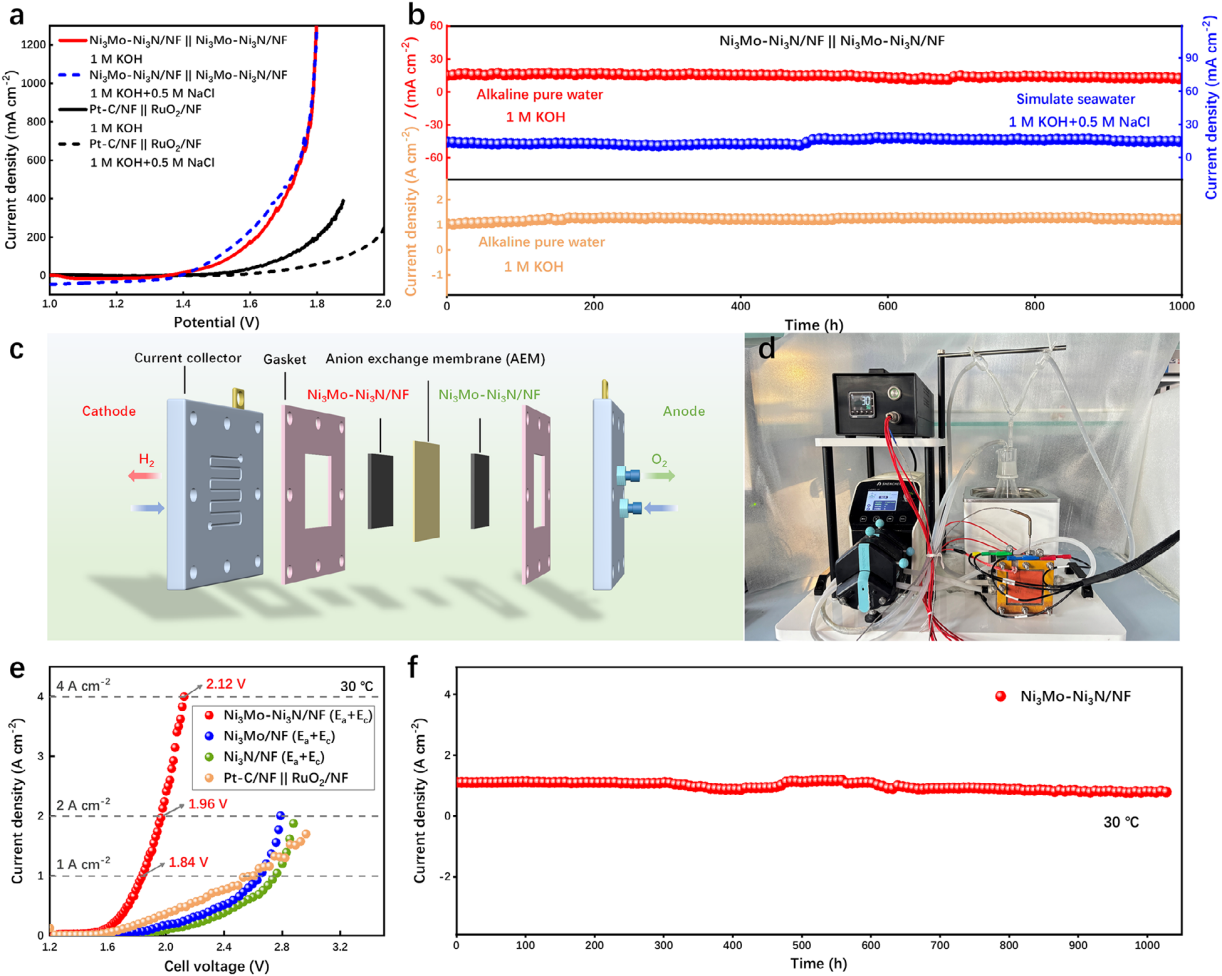
(a) LSV curves and (b) *i–t* tests of Ni_3_Mo–Ni_3_N/NF in different voltages and electrolytes. (c) Schematic illustration of AEMWE. (d) The assembled AEMWE device in the laboratory. (e) Polarization curves for AEMWE. (f) Stability test at 1 A cm^−2^ for 1000 h under room temperature.

Based on the exceptional bifunctional performance of the Ni_3_Mo–Ni_3_N/NF, an alkaline electrolyzer has been constructed by using Ni_3_Mo–Ni_3_N/NF serving as both the cathode and anode. The Ni_3_Mo–Ni_3_N/NF || Ni_3_Mo–Ni_3_N/NF electrode exhibits remarkable water splitting performance in 1 M KOH, as illustrated in Figure 5a, requiring only 1.39 V at 10 mA cm^−2^, outperforming that of Pt‐C/NF || RuO_2_/NF (1.49 V). Moreover, the water splitting voltages are merely 1.73 and 1.79 V at 500 and 1000 mA cm^−2^, respectively. Meanwhile, the Ni_3_Mo–Ni_3_N/NF || Ni_3_Mo–Ni_3_N/NF electrode exhibits exceptional OWS stability, maintaining consistent reactivity for more than 1000 h at current densities of 10 and 1000 mA cm^−2^ in 1 M KOH electrolyte (Figure 5b). Additionally, the practicability of the Ni_3_Mo–Ni_3_N/NF catalyst was further investigated by assembling an electrolyzer in simulated seawater. The results suggest that the performance of the Ni_3_Mo–Ni_3_N/NF in simulated seawater remains largely unaffected, and it can also operate steadily for 1000 h at 10 mA cm^−2^ (Figure 5b). In comparison with recent studies, the Ni_3_Mo–Ni_3_N/NF exhibits remarkable OWS activity and stability (Figure S18; Table S16).

To explore the application potential of Ni_3_Mo–Ni_3_N/NF, we constructed an anion exchange membrane water electrolyzer (AEMWE) and evaluated its performance in the AEM system, as shown in Figure 5c. The Ni_3_Mo–Ni_3_N/NF served as both the anode and cathode of the AEM simultaneously, and subsequently tested at ambient temperature using a 1 M KOH feed solution (Figure 5d). The LSV curve in Figure 5e shows that a voltage of 1.84 V can achieve a current density of 1 A cm^−2^, and only 2.12 V is needed to reach 4 A cm^−2^. Furthermore, a stability test was performed under 1 A cm^−2^, revealing that the electrolyzer maintained stable operation at room temperature for 1000 h (Figure 5f). The results demonstrate that the Ni_3_Mo–Ni_3_N/NF exhibits promising potential for commercial implementation in AEMWE.

**FIGURE 6:**
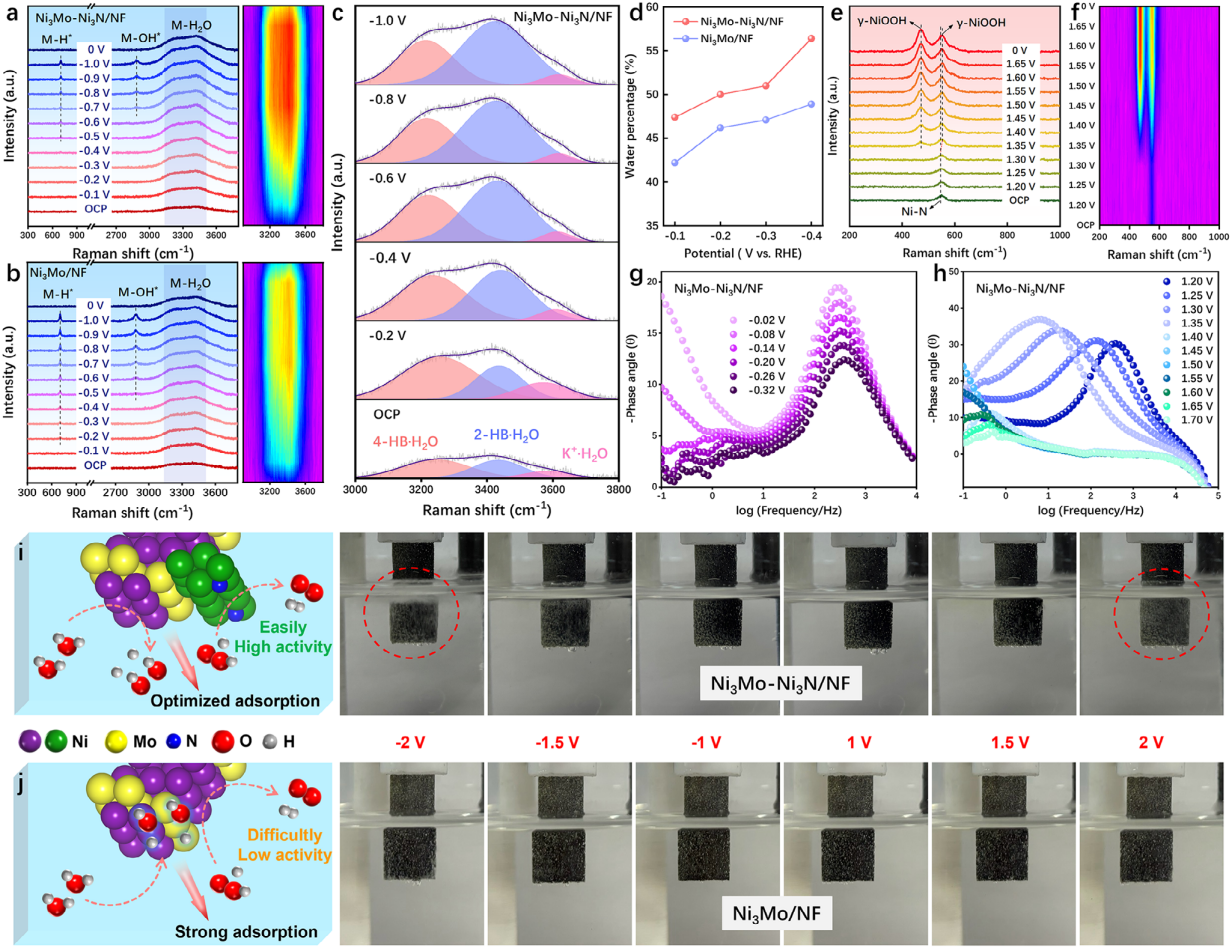
In situ Raman spectra and corresponding contour plots of (a) Ni_3_Mo–Ni_3_N/NF and (b) Ni_3_Mo/NF during HER. (c) In situ Raman spectroscopy for the O─H stretching mode from the interfacial water. (d) Potential dependence of the ratio of the 2‐HB·H_2_O peaks about interfacial water. (e) In situ Raman spectra and (f) corresponding contour plots of Ni_3_Mo–Ni_3_N/NF during OER. EIS Bode plots of Ni_3_Mo–Ni_3_N/NF during (g) HER and (h) OER. Photos of bubbles appearing on the catalyst surface when different voltages are applied, and schematic diagrams about the underlying mechanism of (i) Ni_3_Mo–Ni_3_N/NF and (j) Ni_3_Mo/NF.

The intrinsic catalytic mechanism of the highly active catalyst was further investigated through in situ tests. HER in situ Raman testing was collected in the potential range from −0.1 to −1.0 V (versus Hg/HgO), including both the non‐Faradaic current region and HER region. The in situ Raman spectra and corresponding contour maps of Ni_3_Mo–Ni_3_N/NF and Ni_3_Mo/NF are depicted in Figure 6a,b, respectively. During the HER process, the metal site acts as an adsorption site for both H* and OH* reaction intermediates. Consequently, both Ni_3_Mo–Ni_3_N/NF and Ni_3_Mo/NF catalysts exhibit distinct peaks in metal site adsorption at M─H^*^(701.7 cm^−1^) and M─OH^*^(2881.1 cm^−1^), along with interfacial water absorption peaks ranging from 3160 to 3460 cm^−1^. The disparity lies in the fact that the initial peak potentials pertaining to the M─H* and M─OH* peaks of Ni_3_Mo–Ni_3_N/NF catalysts are −0.5 and −0.7 V, respectively, which surpass those of Ni_3_Mo/NF (−0.2 and −0.5 V). Furthermore, the intensities of these two peaks in Ni_3_Mo–Ni_3_N/NF are comparatively weaker. The results indicate that the Ni_3_Mo/NF exhibits enhanced adsorption capabilities towards H* and OH* reaction intermediates, which in turn hinders their desorption and subsequent reactions. However, the formation of a heterojunction weakens the adsorption strength of the intermediate and increases the catalytic rate. The internal reason may be that the formation of a heterojunction weakens the strong adsorption effect of Ni_3_Mo alloy on the intermediate, and an optimal adsorption strength is more conducive to subsequent H_2_ and O_2_ generation reactions, thereby resulting in higher catalytic activity. Furthermore, the peak strength of interfacial water in Ni_3_Mo–Ni_3_N/NF surpasses that in Ni_3_Mo/NF, suggesting that the formation of heterojunction facilitates the adsorption of surface water. To further elucidate the effect of the heterojunction on interfacial water, we performed quantitative peak decomposition and fitting on the interfacial water signals in the in situ Raman spectra of Ni_3_Mo–Ni_3_N/NF and Ni_3_Mo/NF catalysts. In Figure 6c and Figure S19a, the broad stretching vibrational band observed in the range of 3000–3800 cm^−1^ is assigned to the stretching mode of H_2_O. It was decomposed into three O‒H stretching modes of interfacial water via Gaussian fitting. The peaks at approximately 3200, 3400, and 3600 cm^−1^ correspond to the symmetric stretching vibration of strong H‐bonded water (4‐HB·H_2_O), the asymmetric stretching vibration of weak H‐bonded water (2‐HB·H_2_O), and free water (K+·H_2_O), respectively [43, 44]. Among these species, the 4‐HB·H_2_O requires high energy for proton transfer due to its overly strong hydrogen‐bonding network connectivity. In contrast, free water exhibits low proton transfer efficiency as a result of weak adsorption and poor network connectivity [45, 46]. With the negative voltage increases, the 4‐HB·H_2_O species on the Ni_3_Mo/NF catalyst exhibits the largest Stark tuning rate (37.8 cm^−1^/V) while having the highest quantity (Figure S19b,c). This indicates that the hydrogen‐bonding network of interfacial water on the Ni_3_Mo/NF surface is more rigid, which implies that a higher energy input is required to drive the reorientation of interfacial water for charge transfer. Nevertheless, the 2‐HB·H_2_O, which is beneficial to catalytic activity, gradually becomes dominant on Ni_3_Mo–Ni_3_N/NF with the increase in negative voltage (Figure 6d). This indicates that the formation of heterojunctions facilitates the restructuring of the interfacial hydrogen‐bonding network. An optimally structured network, in turn, promotes charge transfer and enhances the progression of the catalytic reaction.

The OER in situ Raman spectra and corresponding contour plots of Ni_3_Mo–Ni_3_N/NF in the potential range from 1.20 to 1.65 V (versus Hg/HgO) were employed to study the reconstruction process (Figure 6e,f). The characteristic peak of Ni─N at 545 cm^−1^ persists from the open‐circuit potential (OCP) to higher voltages, indicating that the Ni_3_N phase does not fully reconstruct during the OER process [47]. Upon increasing the potential to 1.35 V, two new peaks emerge at 477 and 559 cm^−1^. These can be attributed to the characteristic E_g_ and A_1g_ bands of NiOOH, respectively [48, 49]. The intensity of these peaks gradually increases with rising voltage, suggesting that surface reconstruction occurs at 1.35 V, leading to the formation of the active species NiOOH. This conclusion aligns with the findings from HR‐TEM and XPS analyses (Figures S20 and S21).

In situ electrochemical impedance spectroscopy (EIS) under alkaline conditions enables further investigation of the detailed hydrolysis kinetics of catalysts during the HER. The low‐frequency response reflects HER kinetics, while the high‐frequency response characterizes the electrolyte–electrocatalyst interfacial behavior [50, 51]. Both Ni_3_Mo–Ni_3_N/NF and Ni_3_Mo/NF exhibit reduced phase angles with peak shifts toward higher frequencies in the low‐frequency region (Figure 6g; Figure S22a), demonstrating decreased Faradaic resistance and accelerated surface reaction kinetics under reduced potentials. In the high‐frequency region, Ni_3_Mo/NF shows no distinct peaks across various potentials, indicating stable electrolyte–electrocatalyst interfaces and indirectly confirming its strong adsorption of intermediates during HER, which hinders subsequent reactions. Conversely, Ni_3_Mo–Ni_3_N/NF displays pronounced peaks in the high‐frequency region after heterojunction formation, suggesting that the incorporation of Ni_3_N improves interfacial behavior, consistent with in situ Raman analysis. Notably, Ni_3_N/NF exhibits substantial phase angles in the high‐frequency region with minimal voltage‐dependent variations in the low‐frequency region, indicating sluggish HER kinetics (Figure S22b). Furthermore, in situ EIS analysis of OER offers an understanding of the catalytic kinetic properties and the adsorption/desorption dynamics of reaction intermediates on electrode surfaces. The characteristics of the oxidation reaction inside the electrode are displayed in the high‐frequency range, while OER is related to the response in the low‐frequency range [52, 53]. As illustrated in Figure 6h and Figure S22c, the high‐frequency phase angles for Ni_3_Mo–Ni_3_N/NF and Ni_3_Mo/NF catalysts disappear at a voltage of 1.40 V, indicating the generation of highly conductive metallic active species. This suggests that Ni_3_Mo–Ni_3_N/NF and Ni_3_Mo/NF form active hydroxide species at 1.40 V or higher voltages. Simultaneously, phase angles emerge in the low‐frequency region, gradually decreasing and shifting towards the mid‐frequency range, signifying the occurrence of the OER at 1.40 V and higher potentials, which aligns with the previously mentioned onset potential of OER. However, the low‐frequency phase angle of Ni_3_Mo–Ni_3_N/NF is lower than that of Ni_3_Mo/NF, showing that the adsorption strength of intermediates during the OER process is weaker for Ni_3_Mo‐Ni_3_N/NF, resulting in a lower electron transfer barrier. This demonstrates that the formation of a heterojunction mitigates the stronger intermediate adsorption strength of Ni_3_Mo/NF. In contrast, the in situ EIS spectrum of Ni_3_N/NF (Figure S22d) does not exhibit significant phase angles in the high‐frequency region, suggesting that the catalyst undergoes minimal reconstruction under applied voltage. Additionally, the substantial phase angle in the low‐frequency region for OER indicates the generation of minimal high‐valent active species and a significant electron transfer barrier. Finally, varying voltages are applied to the catalysts, and the resulting rate of bubble production on their surface is observed. The photos and schematic diagrams in Figure 6i,j depict the internal mechanism of Ni_3_Mo–Ni_3_N/NF and Ni_3_Mo/NF catalysts reacting at different voltages, respectively. The application of different voltages to the catalysts reveals a significant increase in the number of bubbles on the Ni_3_Mo–Ni_3_N/NF surface compared to the Ni_3_Mo/NF surface, particularly at −2 and 2 V, where a substantial amount of bubbles are generated on the Ni_3_Mo–Ni_3_N/NF surface. This suggests that the presence of Ni_3_N can effectively enhance the catalytic reaction rate, facilitating the production of H_2_ and O_2_.

**FIGURE 7:**
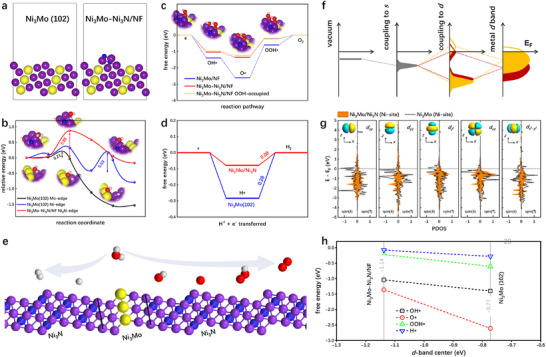
(a) Slab models for Ni_3_Mo (102) and Ni_3_Mo‐Ni_3_N/NF. (b) The energy barrier for breaking the H‐OH bond on the Mo and Ni edges of Ni_3_Mo (102) and the Ni_3_N edge of Ni_3_Mo‐Ni_3_N/NF. Free energy diagrams for (c) OER at equilibrium potential (1.23 V), and (d) HER at zero voltage on the Ni edge of Ni_3_Mo (102) and the Ni_3_N edge of Ni_3_Mo‐Ni_3_N/NF. (e) Schematic representation of the water splitting mechanism on Ni_3_Mo‐Ni_3_N/NF. (f) Schematic representation of the coupling between the adsorbate state and the metal *d*‐band. (g) Projected density of states (PDOS) of the *d*‐bands for Ni atoms in the Ni edge of Ni_3_Mo (102) and the Ni_3_N edge of Ni_3_Mo‐Ni_3_N/NF. The horizontal line indicates the Fermi level. (h) Free energy for OER and HER intermediates as a function of the Ni *d*‐band center (spin up).

DFT calculations had been implemented to unravel the underlying mechanism behind the boosted OER and HER properties on the Ni_3_Mo–Ni_3_N/NF catalyst. Based on the experimental HR‐TEM and SAED, a slab model consisting of the Ni_3_N motif anchored on the stepped Ni_3_Mo (102) surface is constructed to simulate the structure of Ni_3_Mo–Ni_3_N/NF (Figure 7a; Figures S23 and S24). The energy barrier for H_2_O dissociation into H* (* indicates an adsorbed species) and OH* intermediates is estimated through conducting climbing image nudged elastic band (CI‐NEB) simulations. As shown in Figure 7b, the barrier for breaking the H‐OH bond in H_2_O is calculated as 0.21, 0.63, and 0.88 eV on the Mo‐edge of Ni_3_Mo (102), Ni‐edge of Ni_3_Mo (102), and Ni_3_N‐site of Ni_3_Mo‐Ni_3_N/NF, respectively, indicating that the H_2_O dissociation can be achieved on the Ni_3_Mo (102) surface. The relatively low activation barrier for H_2_O dissociation on the Mo‐edge of Ni_3_Mo (102) can be attributed to the strong adsorption of H* and OH*, resulting in a low reaction energy and thus a low activation energy following the Brønsted‐Evans‐Polanyi relation. However, the strong adsorption of H* and OH* intermediates results in sluggish kinetics for both the subsequent OER and HER. In Figure 7c,d, the relative free energies of OH*, O* (for OER), and H* (for HER) on Ni_3_Mo (102) are much more negative than zero, i.e., the optimal value to minimize the reaction overpotential, suggesting that the generation of O_2_ and H_2_ would be difficult if catalyzed solely by the Ni_3_Mo (102) surface. In contrast, we find that the adsorption strength of OH*, O*, and H* can be dramatically weakened on the Ni_3_N‐site of Ni_3_Mo‐Ni_3_N/NF, resulting in low reaction free energies for the overpotential‐determining steps, e.g., O* + OH^−^ → OOH* for OER and H* + H^+^ + e^−^ → H_2_ for HER (Figure 7c,d). Besides, we constructed a model of Ni_3_Mo–Ni_3_N/NF with surface‐adsorbed ‐OOH (Figure S25) and calculated the Gibbs free energy of the OER process for this model (Figure 7c). The results show that the catalyst with surface‐adsorbed ‐OOH exhibits more favorable intermediate adsorption energies, thereby demonstrating faster OER reaction kinetics. These results illustrate that the enhanced OER and HER activities on the Ni_3_Mo–Ni_3_N/NF catalyst stem from the synergy between Ni_3_Mo and Ni_3_N. In particular, the dissociation of H_2_O can be achieved on the Ni_3_Mo surface while the subsequent evolution reactions are catalyzed by the adjacent Ni_3_N sites (Figure 7e), which is in line with the results obtained from in situ Raman and in situ EIS analysis.

The discrepancy in the adsorption strength of OH*, O*, and H* intermediates on the Ni_3_Mo (102) and Ni_3_Mo–Ni_3_N/NF surfaces can be rationalized through the *d*‐band theory [54]. Specifically, within the framework of the *d*‐band theory, the interaction strength between a metal and reaction intermediates is governed by the position of the metal *d*‐band (Figure 7f). If the *d*‐band is lower in energy (keep away from the Fermi level), the split between the bonding and antibonding states will be decreased [55]. As a result, the filling of the adsorbate‐metal antibonding states weakens the adsorption strength. As shown in Figure 7g, we find that the band centers of the five Ni *d* orbitals in the Ni_3_N‐sites of Ni_3_Mo–Ni_3_N/NF are all lower than that in the Ni‐sites of Ni_3_Mo (102), clarifying the weakened adsorption of the intermediates during OER and HER [6]. Conversely, the relatively high *d*‐band centers are responsible for the strong adsorption of the OH*, O*, and H* intermediates on Ni_3_Mo (102) (Figure 7h). These findings align with the experimental observations from XPS and UPS analyses, underscoring the crucial role of the *d*‐electronic structure in modulating the OER and HER properties of the Ni_3_Mo–Ni_3_N/NF catalyst. In particular, mismatching the metal *d*‐bands by heterojunction construction could be an effective means to decouple the adsorption energy scaling relationships between reaction intermediates and thus activate the metal centers for electrocatalysis [56].

## 3 Conclusion

In summary, a heterojunction catalyst composed of Ni_3_Mo and Ni_3_N was successfully synthesized in this work, which demonstrates exceptional HER and OER performances. The Ni_3_Mo–Ni_3_N/NF achieves ultra‐low overpotentials of 15 mV (HER) and 155 mV (OER) at 10 mA cm^−2^, and just 228 mV (HER) and 459 mV (OER) at 1 A cm^−2^. Furthermore, the Ni_3_Mo–Ni_3_N/NF also demonstrates remarkable activity and stability for OWS, requiring low applied voltages of only 1.39 and 1.79 V to reach 10 and 1000 mA cm^−2^, respectively, while maintaining virtually unchanged properties in simulated seawater solutions. Moreover, the Ni_3_Mo–Ni_3_N/NF demonstrates outstanding performance in AEMWE, reaching 4 A cm^−2^ at a low voltage of 2.12 V while maintaining stable operation for 1000 h at 1 A cm^−2^ under room temperature. In situ Raman and DFT theoretical calculations have confirmed that the presence of Ni_3_Mo and Ni_3_N components in the heterojunction contributes to OWS. This indicates that the formation of heterojunctions leads to a mismatch in the *d*‐band center of the metal sites, which facilitates precise regulation of intermediate adsorption and consequently enhances electrocatalytic activity.

## Conflicts of Interest

The authors declare no conflicts of interest.

## Supporting information




**Supporting File**: advs73793‐sup‐0001‐SuppMat.docx.

## Data Availability

The data that support the findings of this study are available in the supplementary material of this article.

## References

[1] Y. Sun , H. Liao , J. Wang , et al., “Covalency Competition Dominates the Water Oxidation Structure–Activity Relationship on Spinel Oxides,” Nature Catalysis 3 (2020): 554–563, 10.1038/s41929-020-0465-6.

[2] Y. Jia , L. Zhang , L. Zhuang , et al., “Identification of Active Sites for Acidic Oxygen Reduction on Carbon Catalysts with and without Nitrogen Doping,” Nature Catalysis 2 (2019): 688–695, 10.1038/s41929-019-0297-4.

[3] Y. Ma , M. Chen , H. Geng , et al., “Synergistically Tuning Electronic Structure of Porous *β*‐Mo_2_C Spheres by Co Doping and Mo‐Vacancies Defect Engineering for Optimizing Hydrogen Evolution Reaction Activity,” Advanced Functional Materials 30 (2020): 2000516, 10.1002/adfm.202000561.

[4] X. Zhang , A. Wu , D. Wang , et al., “Fine‐Tune the Electronic Structure in Co‐Mo Based Catalysts to Give Easily Coupled HER and OER Catalysts for Effective Water Splitting,” Applied Catalysis B: Environmental 328 (2023): 122474, 10.1016/j.apcatb.2023.122474.

[5] J. Wang , N. Zang , C. Xuan , B. Jia , W. Jin , and T. Ma , “Self‐Supporting Electrodes for Gas‐Involved Key Energy Reactions,” Advanced Functional Materials 31 (2021): 2104620, 10.1002/adfm.202104620.

[6] P. Kuang , Z. Ni , B. Zhu , Y. Lin , and J. Yu , “Modulating the d‐Band Center Enables Ultrafine Pt_3_ Fe Alloy Nanoparticles for pH‐Universal Hydrogen Evolution Reaction,” Advanced Materials 35 (2023): 2303030, 10.1002/adma.202303030. 37392140

[7] X. Zhao , B. Pattengale , D. Fan , et al., “Mixed‐Node Metal–Organic Frameworks as Efficient Electrocatalysts for Oxygen Evolution Reaction,” ACS Energy Letters 3 (2018): 2520–2526, 10.1021/acsenergylett.8b01540.

[8] P. Zhou , P. Niu , J. Liu , et al., “Anodized Steel: the Most Promising Bifunctional Electrocatalyst for Alkaline Water Electrolysis in Industry,” Advanced Functional Materials 32 (2022): 2202068, 10.1002/adfm.202202068.

[9] G. Yang , Y. Jiao , H. Yan , et al., “Interfacial Engineering of MoO_2_ ‐FeP Heterojunction for Highly Efficient Hydrogen Evolution Coupled With Biomass Electrooxidation,” Advanced Materials 32 (2020): 2000455, 10.1002/adma.202000455. 32173914

[10] N. Guo , H. Xue , R. Ren , et al., “S‐Block Potassium Single‐atom Electrocatalyst With K−N_4_ Configuration Derived From K + /Polydopamine for Efficient Oxygen Reduction,” Angewandte Chemie International Edition 62 (2023): 202312409, 10.1002/anie.202312409. 37681482

[11] J. Sun , H. Xue , N. Guo , et al., “Synergetic Metal Defect and Surface Chemical Reconstruction Into NiCo_2_S_4_/ZnS Heterojunction to Achieve Outstanding Oxygen Evolution Performance,” Angewandte Chemie International Edition 60 (2021): 19435–19441, 10.1002/anie.202107731. 34153176

[12] D. Liu , H. Ai , J. Li , et al., “Surface Reconstruction and Phase Transition on Vanadium–Cobalt–Iron Trimetal Nitrides to Form Active Oxyhydroxide for Enhanced Electrocatalytic Water Oxidation,” Advanced Energy Materials 10 (2020): 2002464, 10.1002/aenm.202002464.

[13] Q. Niu , M. Yang , D. Luan , N. W. Li , L. Yu , and X. W. Lou , “Construction of Ni‐Co‐Fe Hydr(oxy)oxide@Ni‐Co Layered Double Hydroxide Yolk‐Shelled Microrods for Enhanced Oxygen Evolution,” Angewandte Chemie International Edition 61 (2022): 202213049, 10.1002/anie.202213049. 36218244

[14] M. Liu , K. A. Min , B. Han , and L. Y. S. Lee , “Interfacing or Doping? Role of Ce in Highly Promoted Water Oxidation of NiFe‐Layered Double Hydroxide,” Advanced Energy Materials 11 (2021): 2101281, 10.1002/aenm.202101281.

[15] X. Zhang , F. Yan , X. Ma , et al., “Regulation of Morphology and Electronic Structure of FeCoNi Layered Double Hydroxides for Highly Active and Stable Water Oxidization Catalysts,” Advanced Energy Materials 11 (2021): 2102141, 10.1002/aenm.202102141.

[16] J. Zhou , Y. Hu , Y.‐C. Chang , et al., “In Situ Exploring of the Origin of the Enhanced Oxygen Evolution Reaction Efficiency of Metal(Co/Fe)–Organic Framework Catalysts Via Postprocessing,” ACS Catalysis 12 (2022): 3138–3148, 10.1021/acscatal.1c05532.

[17] S. Shaik , J. Kundu , Y. Yuan , et al., “Recent Progress and Perspective in Pure Water‐Fed Anion Exchange Membrane Water Electrolyzers,” Advanced Energy Materials 14 (2024): 2401956, 10.1002/aenm.202401956.

[18] F. Bao , Z. Yang , Y. Yuan , et al., “Synergistic Cascade Hydrogen Evolution Boosting via Integrating Surface Oxophilicity Modification With Carbon Layer Confinement,” Advanced Functional Materials 32 (2021): 2108991, 10.1002/adfm.202108991.

[19] L. Gao , F. Bao , X. Tan , et al., “Engineering a Local Potassium Cation Concentrated Microenvironment toward the Ampere‐Level Current Density Hydrogen Evolution Reaction,” Energy & Environmental Science 16 (2023): 285–294, 10.1039/D2EE02836K.

[20] J. Zhang , T. Wang , P. Liu , et al., “Efficient Hydrogen Production on MoNi_4_ Electrocatalysts With Fast Water Dissociation Kinetics,” Nature Communications 8 (2017): 15437, 10.1038/ncomms15437. PMC5442356 28513620

[21] X. Shi , X. Zheng , H. Wang , et al., “Hierarchical Crystalline/Amorphous Heterostructure MoNi/NiMoO_x_ for Electrochemical Hydrogen Evolution With Industry‐Level Activity and Stability,” Advanced Functional Materials 33 (2023): 2307109, 10.1002/adfm.202307109.

[22] C. Pan , Z. Mao , X. Yuan , H. Zhang , L. Mei , and X. Ji , “Heterojunction Nanomedicine,” Advanced Science 9 (2022): 2105747, 10.1002/advs.202105747. 35174980 PMC9008793

[23] Y. Yan , Z. Zeng , M. Huang , and P. Chen , “Van Der Waals Heterojunctions for Catalysis,” Materials Today Advances 6 (2020): 100059, 10.1016/j.mtadv.2020.100059.

[24] D. Zheng , L. Yu , W. Liu , et al., “Structural Advantages and Enhancement Strategies of Heterostructure Water‐Splitting Electrocatalysts,” Cell Reports Physical Science 2 (2021): 100443, 10.1016/j.xcrp.2021.100443.

[25] S. Jiao , X. Fu , and H. Huang , “Descriptors for the Evaluation of Electrocatalytic Reactions: D‐Band Theory and Beyond,” Advanced Functional Materials 32 (2022): 2107651, 10.1002/adfm.202107651.

[26] Y. Li , Y. Jiao , H. Yan , et al., “Mo−Ni‐Based Heterojunction With Fine‐Customized d‐Band Centers for Hydrogen Production Coupled With Benzylamine Electrooxidation in Low Alkaline Medium,” Angewandte Chemie International Edition 62 (2023): 202306640, 10.1002/anie.202306640. 37312604

[27] X. Wu , Z. Shao , Q. Zhu , et al., “Tuning the *d*‐Band Center of Co_3_O_4_ via Octahedral and Tetrahedral Codoping for Oxygen Evolution Reaction_14_O_4_ via Octahedral and Tetrahedral Codoping for Oxygen Evolution Reaction,” ACS Catalysis (2024): 5888–5897, 10.1021/acscatal.3c06256.

[28] X. Sun , L. Sun , G. Li , et al., “Phosphorus Tailors the d ‐Band Center of Copper Atomic Sites for Efficient CO_2_ Photoreduction Under Visible‐Light Irradiation,” Angewandte Chemie International Edition 61 (2022): 202207677, 10.1002/anie.202207677. 35801835

[29] X. Xu , H. Liao , L. Huang , et al., “Surface Reconstruction and Directed Electron Transport in NiSe_2_/MoSe_2_ Mott‐Schottky Heterojunction Catalysts Promote Urea‐Assisted Water Splitting,” Applied Catalysis B: Environmental 341 (2024): 123312, 10.1016/j.apcatb.2023.123312.

[30] H. Jia , H. Wang , F. Yan , H. Zhang , Z. Li , and J. Wang , “Unravelling Electrocatalytic Concerted Diatomic‐Ensembles over Superior Hydrogen‐Evolution Array Structured by NiMo/Mo_2_N Heteronanojunctions,” Applied Catalysis B: Environmental 343 (2024): 123362, 10.1016/j.apcatb.2023.123362.

[31] X. Du , X. Lei , L. Zhou , et al., “Bimetallic Ni and Mo Nitride as an Efficient Catalyst for Hydrodeoxygenation of Palmitic Acid,” ACS Catalysis 12 (2022): 4333–4343, 10.1021/acscatal.1c05847.

[32] H. Hu , Z. Zhang , Y. Zhang , et al., “An Ultra‐Low Pt Metal Nitride Electrocatalyst for Sustainable Seawater Hydrogen Production,” Energy & Environmental Science 16 (2023): 4584–4592, 10.1039/D3EE01541F.

[33] C. Rao , H. Wang , K. Chen , et al., “Hybrid Acid/Base Electrolytic Cell for Hydrogen Generation and Methanol Conversion Implemented by Bifunctional Ni/MoN Nanorod Electrocatalyst,” Small 20 (2023): 2303300, 10.1002/smll.202303300. 37840438

[34] J.‐T. Ren , L. Chen , H.‐Y. Wang , et al., “Synergistic Activation of Crystalline Ni_2_P and Amorphous NiMoOx for Efficient Water Splitting at High Current Densities,” ACS Catalysis 13 (2023): 9792–9805, 10.1021/acscatal.3c01885.

[35] Z. Zhu , L. Luo , Y. He , et al., “High‐Performance Alkaline Freshwater and Seawater Hydrogen Catalysis by Sword‐Head Structured Mo_2_N‐Ni_3_Mo_3_N Tunable Interstitial Compound Electrocatalysts,” Advanced Functional Materials 34 (2023): 2306061, 10.1002/adfm.202306061.

[36] M. Ning , Y. Wang , L. Wu , et al., “Hierarchical Interconnected NiMoN With Large Specific Surface Area and High Mechanical Strength for Efficient and Stable Alkaline Water/Seawater Hydrogen Evolution,” Nano‐Micro Letters 15 (2023): 157, 10.1007/s40820-023-01129-y. 37336833 PMC10279610

[37] Z. Sun , Y. Wang , L. Zhang , et al., “Simultaneously Realizing Rapid Electron Transfer and Mass Transport in Jellyfish‐Like Mott–Schottky Nanoreactors for Oxygen Reduction Reaction,” Advanced Functional Materials 30 (2020): 1910482, 10.1002/adfm.201910482.

[38] J. Wang , B. Huang , Y. Ji , et al., “A General Strategy to Glassy M‐Te (M = Ru, Rh, Ir) Porous Nanorods for Efficient Electrochemical N_2_ Fixation,” Advanced Materials 32 (2020): 1907112, 10.1002/adma.201907112. 32020715

[39] J. Wang , S. Xin , Y. Xiao , et al., “Manipulating the Water Dissociation Electrocatalytic Sites of Bimetallic Nickel‐Based Alloys for Highly Efficient Alkaline Hydrogen Evolution,” Angewandte Chemie International Edition 61 (2022): 202202518, 10.1002/ange.202202518. 35441413

[40] K. Yan , T. A. Maark , A. Khorshidi , V. A. Sethuraman , A. A. Peterson , and P. R. Guduru , “The Influence of Elastic Strain on Catalytic Activity in the Hydrogen Evolution Reaction,” Angewandte Chemie 128 (2016): 6283–6289, 10.1002/ange.201508613. 27079940

[41] Y. Zhang , Y. Zhang , B. Tian , H. Li , Z. Zeng , and D. Ho , “D‐Band Center Optimization of Iron Carbide via Cr Substitution for Enhanced Alkaline Hydrogen Evolution,” Materials Today Energy 29 (2022): 101133, 10.1016/j.mtener.2022.101133.

[42] B. Hammer , “Special Sites at Noble and Late Transition Metal Catalysts,” Topics in Catalysis 37 (2006): 3–16, 10.1007/s11244-006-0004-y.

[43] K. Ji , S. Wang , S. Yao , et al., “Modeling Carbon‐Free Energy Conversion Systems: Enhanced Hydrazine‐Assisted Hydrogen Production With Dual‐Electric‐Field Effect on Needle‐Like Ru/CoP Catalysts,” Energy & Environmental Science 18 (2025): 4764–4774, 10.1039/D4EE05691D.

[44] Z. Qin , J. Li , Q. Wu , et al., “Topologically Close‐Packed Frank‐Kasper C15 Phase Intermetallic Ir Alloy Electrocatalysts Enables High‐Performance Proton Exchange Membrane Water Electrolyzer,” Advanced Materials 36 (2024): 2412541, 10.1002/adma.202412541. 39350447

[45] S. Zhou , W. Cao , L. Shang , et al., “Facilitating Alkaline Hydrogen Evolution Kinetics via Interfacial Modulation of Hydrogen‐Bond Networks by Porous Amine Cages,” Nature Communications 16 (2025): 1849, 10.1038/s41467-025-56962-z. PMC11845474 39984442

[46] S. Wang , T. Jiang , Y. Hao , et al., “Unveiling the Cation Dependence in Alkaline Hydrogen Evolution by Differently‐Charged Ruthenium/Molybdenum Sulfide Hybrids,” Advanced Materials 36 (2024): 2410422, 10.1002/adma.202410422. 39300910

[47] X. Ding , R. Jiang , J. Wu , et al., “Ni_3_ N–CeO_2_ Heterostructure Bifunctional Catalysts for Electrochemical Water Splitting,” Advanced Functional Materials 33 (2023): 2306786, 10.1002/adfm.202306786.

[48] Y. Li , J. Cai , J. Zhang , et al., “An Unexpected Electrochemical Performance Enabled by In Situ Formed Quasi‐Metal‐Semiconductor Heterojunction With Innumerous P‐Type Anti‐Barrier Layer,” Advanced Energy Materials 13 (2023): 2204114, 10.1002/aenm.202204114.

[49] K. Deng , X. Liu , P. Liu , X. Lv , W. Tian , and J. Ji , “Enhanced Adsorption Kinetics and Capacity of a Stable CeF_3_ @Ni_3_ N Heterostructure for Methanol Electro‐Reforming Coupled With Hydrogen Production,” Angewandte Chemie International Edition 64 (2024): 202416763, 10.1002/anie.202416763. 39523460

[50] C. Ye , S. Yao , X. Xiao , et al., “Interfacial Electric Field‐Mediated Stabilization of Unsaturated RuO_2– x_ Clusters on MoO_3_ –Ni(OH)_2_ Heterostructure for Enhanced pH‐Universal Hydrogen Evolution,” ACS Nano 19 (2025): 26782–26790, 10.1021/acsnano.5c06948. 40662913

[51] X. Chen , M. Bi , Q. Yan , et al., “Ce Single Atom‐Engineered Amorphous/Crystalline Nanosheets for Enhanced Alkaline Water Electrolysis,” Advanced Materials 37 (2025): 08893, 10.1002/adma.202508893. PMC12710578 40745927

[52] S. Zhou , H. He , J. Li , et al., “Regulating the Band Structure of Ni Active Sites in Few‐Layered Nife‐LDH by In Situ Adsorbed Borate for Ampere‐Level Oxygen Evolution,” Advanced Functional Materials 34 (2023): 2313770, 10.1002/adfm.202313770.

[53] Z. Li , Y. Feng , Y. Li , et al., “Fabrication of Bi/Sn Bimetallic Electrode for High‐Performance Electrochemical Reduction of Carbon Dioxide to Formate,” Chemical Engineering Journal 428 (2022): 130901, 10.1016/j.cej.2021.130901.

[54] B. Hammer and J. K. Norskov , “Why Gold is the Noblest of all the Metals,” Nature 376 (1995): 238–240, 10.1038/376238a0.

[55] F. Xiao , Q. Bao , C. Sun , et al., “D‐Band Center Regulation for Durable Catalysts and Constructing a Robust Hybrid Layer on Li Anode Enable Long‐Life Li‐O_2_ Batteries,” Advanced Energy Materials 14 (2024): 2303766, 10.1002/aenm.202303766.

[56] J. Su , D. Pan , Y. Dong , et al., “Ultrafine Fe_2_C Iron Carbide Nanoclusters Trapped in Topological Carbon Defects for Efficient Electroreduction of Carbon Dioxide,” Advanced Energy Materials 13 (2023): 2204391, 10.1002/aenm.202204391.

