# Supplementary material for: Tailoring Intermediate Adsorption Through d‐band Mismatch Strategy of Heterojunction to Achieve Industrial Overall Water Splitting
Source: Adv Sci (Weinh). 2026 Jan 12;13(17):e21184. doi: 10.1002/advs.202521184 (PMC13042903; doi:10.1002/advs.202521184)
Supplement: Supplementary file 1 — Supporting File: advs73793‐sup‐0001‐SuppMat.docx. [file ADVS-13-e21184-s001.docx]

Supporting Information

**Tailoring Intermediate Adsorption through d-band Mismatch Strategy of Heterojunction to Achieve Industrial Overall Water Splitting**

*Jia Liu, Jiawen Sun, Yi-Ru Hao, Yaqin Chen, Chunhao Li, Le-Le Ma, Jing Sun, Zhonglong Zhao,* Jiangwei Zhang, Hui Xue,* and Qin Wang**

J. Liu, J. Sun, Y.-R. Hao, Y. Chen, C. Li, L.-L. Ma, J. Sun, H. Xue, Q. Wang

College of Chemistry and Chemical Engineering

Inner Mongolia University

Hohhot 010021, China

E-mail: qinwang@imu.edu.cn (Q. Wang); hxue@imu.edu.cn (H. Xue)

Z. Zhao

College of Physical Science and Technology

Inner Mongolia University

Hohhot 010021, China

E-mail: zlzhao@imu.edu.cn

J. Zhang, Q. Wang

College of Energy Materials and Chemistry

Inner Mongolia Key Laboratory of Low Carbon Catalysis

Inner Mongolia University

Hohhot 010021, China

**1. Experimental**

**Materials**

Ni(NO_3_)_2_·6H_2_O was purchased from Shanghai Sinopharm Chemical Reagent Technology Ltd. (NH_4_)_6_Mo_7_O_24_·4H_2_O was purchased from Fuchen (Tianjin) Chemical Reagent Technology Ltd. KOH, 2-Methylimidazole, RuO_2_, and platinum carbon were purchased from Aladdin. Deionized water was used to prepare all solutions.

**Preparation of precursor**

Foam nickel pretreatment: The nickel foam (NF, 2 cm × 3 cm) underwent ultrasonication in 3 M HCl and anhydrous ethanol for a duration of 15 minutes, followed by rinsing with deionized water for subsequent utilization. The mixture of 0.6 mmol Ni(NO_3_)_2_·9H_2_O, 0.4 mmol (NH_4_)_6_Mo_7_O_24_·4H_2_O, and 0.6 mmol 2-Methylimidazole was dissolved in 15 mL of deionized water. After undergoing ultrasound treatment for 10 minutes, the solution completely dissolved and was subsequently transferred into a 50 mL autoclave. The prepared NF was immersed in the aforementioned solution and subjected to heating at a temperature of 160 ℃ for 10 hours. After being cooled to room temperature, the obtained precursor was subjected to multiple washes with deionized water and ethanol. Subsequently, the product was dried in a vacuum oven at 60 °C for 4 hours.

**Preparation of Ni_3_Mo-Ni_3_N/NF**

The prepared precursor (1 cm × 2 cm) was positioned on one side of the porcelain boat, while 0.5 g of melamine was placed on the opposite side. The porcelain boat was placed in the tube furnace with the melamine side facing upstream, and then subjected to nitridation at a temperature of 500 ℃ with a heating rate of 5 °C min^-1^ for a duration of 1 h under an N_2_ atmosphere.

**Preparation of Ni_3_Mo/NF**

1 mmol (NH_4_)_6_Mo_7_O_24_·4H_2_O and 0.6 mmol 2-Methylimidazole were dissolved in 15 mL of deionized water. After 10 minutes of ultrasound, it was fully dissolved and transferred into a 50 mL autoclave. The prepared NF is immersed in the above solution and heated at 160 ℃ for 10 h. After cooling to room temperature, the obtained precursor was washed several times with deionized water and ethanol. The product was then dried in a vacuum oven at 60 °C for 4 h. The Ni_3_Mo/NF was obtained by calcining at 500 ℃ with a heating rate of 5 °C min^-1^ for a duration of 1 h under an N_2_ atmosphere.

**Preparation of Ni_3_N/NF**

0.6 mmol of Ni(NO_3_)_2_·9H_2_O and 0.6 mmol of 2-Methylimidazole were dissolved in 15 mL of deionized water, and after being subjected to ultrasound for 10 minutes, the mixture was completely dissolved and transferred into a 50 mL autoclave. The prepared NF was immersed in the above solution and heated at 160 ℃ for 10 h. After cooling to room temperature, the obtained precursor was washed several times with deionized water and ethanol. The product was then dried in a vacuum oven at 60 °C for 4 h. The nitriding process is consistent with Ni_3_Mo-Ni_3_N/NF.

**Preparation of Pt-C/NF**

5 mg Pt/C was added to a mixed solution of 25 μL of Nafion (5%), 250 μL of anhydrous ethanol solution, and 250 μL of deionized water to form ink. The ink was then dispensed onto the carbon cloth (Geometric Area: 0.25 cm^2^) in increments of 5 μL, with a total of three drops (15 μL in total).

**Preparation of RuO_2_/NF**

5 mg RuO_2_ was added to a mixed solution of 25 μL of Nafion (5%), 250 μL of anhydrous ethanol solution, and 250 μL of deionized water to form ink. The ink was then dispensed onto the carbon cloth (Geometric Area: 0.25 cm^2^) in increments of 5 μL, with a total of three drops (15 μL in total).

**2. Material characterization**

The X-ray diffraction (XRD) patterns were investigated by a PuXi XD3 diffractometer equipped with Cu-Kα radiation. X-ray photoelectron spectra (XPS) were corrected using the C 1s line at 284.6 eV and were used to analyze the surface chemical composition and valence states of the catalysts on an ESCALAB 250 X-ray photoelectron spectrometer. The scanning electron microscopy (SEM) was carried out on a JEOL JSM-6700F scanning electron microscope. The transmission electron microscopy (TEM) and high-resolution transmission electron microscopy (HRTEM) images were obtained on a JEOL JEM-2100F transmission electron microscope system.

**3. XAFS analysis and results**

The acquired EXAFS data were processed according to the standard procedures using the ATHENA module of the Demeter software packages.

The EXAFS spectra were obtained by subtracting the post-edge background from the overall absorption and then normalizing with respect to the edge-jump step. Subsequently, the χ(k) data of were Fourier transformed to real (R) space using a hanning windows (dk=1.0 Å^-1^) to separate the EXAFS contributions from different coordination shells. To obtain the quantitative structural parameters around central atoms, least-squares curve parameter fitting was performed using the ARTEMIS module of Demeter software packages.

The following EXAFS equation was used:

$$\chi\left( k \right)=\sum_{j} \frac{N_{j}S_{0}^{2}F_{j}(k)}{kR_{j}^{2}}\cdot\exp\left[ -2k^{2}\sigma_{j}^{2} \right]\cdot exp[\frac{-2R_{j}}{\lambda(k)}{]\cdot sin[2kR_{j}+\Phi_{j}\left( k \right)]}$$

the theoretical scattering amplitudes, phase shifts and the photoelectron mean free path for all paths calculated. S_0_^2^ is the amplitude reduction factor, F_j_(k) is the effective curved-wave backscattering amplitude, N_j_ is the number of neighbors in the j^th^ atomic shell, R_j_ is the distance between the Xray absorbing central atom and the atoms in the j^th^ atomic shell (backscatterer), λ is the mean free path in Å, ϕ_j_(k) is the phase shift (including the phase shift for each shell and the total central atom phase shift), σ_j_ is the Debye-Waller parameter of the j^th^ atomic shell (variation of distances around the average R^j^). The functions F^j^(k), λ and ϕ_j_(k) were calculated with the ab initio code FEFF10. The additional details for EXAFS simulations are given below.

All fits were performed in the *R* space with *k*-weight of 2 while phase correction was also applied in the first coordination shell to make R value close to the physical interatomic distance between the absorber and shell scatterer. The coordination numbers of model samples were fixed as the nominal values. While the S_0_^2^, internal atomic distances R, Debye-Waller factor σ^2^, and the edge-energy shift Δ were allowed to run freely.

**4. Electrochemical measurements**

The electrochemical properties of the catalysts were tested using a three-electrode system on a Shanghai CHI760E electrochemical workstation. The prepared catalyst was used as the working electrode (working area of 0.5 × 0.5 cm^2^), the carbon rod was used as the counter electrode, and the new saturated calomel electrode was used as the reference electrode. The OER, HER, and OWS are tested by using linear scanning voltammetry (LSV) at a scan rate of 2 mV·s^-1^ in 1.0 M KOH and corrected for the automatic iR compensation (85%). Overall seawater splitting is tested by using linear scanning voltammetry (LSV) at a scan rate of 2 mV·s^-1^ in alkaline seawater (seawater+1.0 M KOH) and corrected for the automatic iR compensation (85%). Cyclic voltammetry (CV) was used to measure the double-layer capacitance (C_dl_) of the catalyst with different scanning rates (10, 20, 30, 40, 50, and 60 mV·s^-1^). Electrochemical impedance spectroscopy (EIS) data are recorded at 0.5 V with an amplitude of 5 mV over a frequency range from 10 Hz to 10^6^ Hz. Using the drainage method to test the Faradaic efficiency of the catalyst. The chemical stability of the catalyst was assessed by a chrono potentiometric durability curve (i-t) in 1 M KOH.

**5. Theoretical calculation**

Spin-polarized DFT calculations were performed using the Vienna ab initio Simulation Package (VASP) with the projector-augmented wave (PAW) pseudopotentials^[1, 2]^. The exchange-correlation interaction was described by using the Perdew-Burke-Ernzerhof (PBE) functional^[3, 4]^. The plane-wave energy cutoff was taken as 400 eV. The Brillouin zone was sampled based on the Monkhorst-Pack scheme with a 3×3×1 k-point mesh^[5]^. The transition state for H_2_O dissociation was determined using the Climbing-Image-Nudged Elastic Band (CI-NEB) method^[6]^. The transition states were searched with six images. The Ni_3_Mo (102) slab model was constructed based on the Ni_3_Mo polycrystalline (space group: *Pmmn*; lattice parameter: a = 5.064, b = 4.228, c = 4.448), consistent with our experimental XRD results. The adjacent slabs were separated by a 15 Å vacuum in the normal direction. The computational hydrogen electrode (CHE) model^[7]^ was employed to calculate the free energy change in each reaction step. To calculate the free energy of intermediates, the corrections for zero-point energy, heat capacity, and entropy were included.

**Figure S1.** XRD spectra of a) various precursor samples and b) final samples of Ni_3_Mo/NF, Ni_3_N/NF, and Ni_3_Mo-Ni_3_N/NF.

**Figure S2.** a-c) SEM images of Ni_3_Mo/NF.

**Figure S3.** a-c) SEM images of Ni_3_N/NF.

**Figure S4.** a-c) SEM images of Ni_3_Mo-Ni_3_N/NF.

**
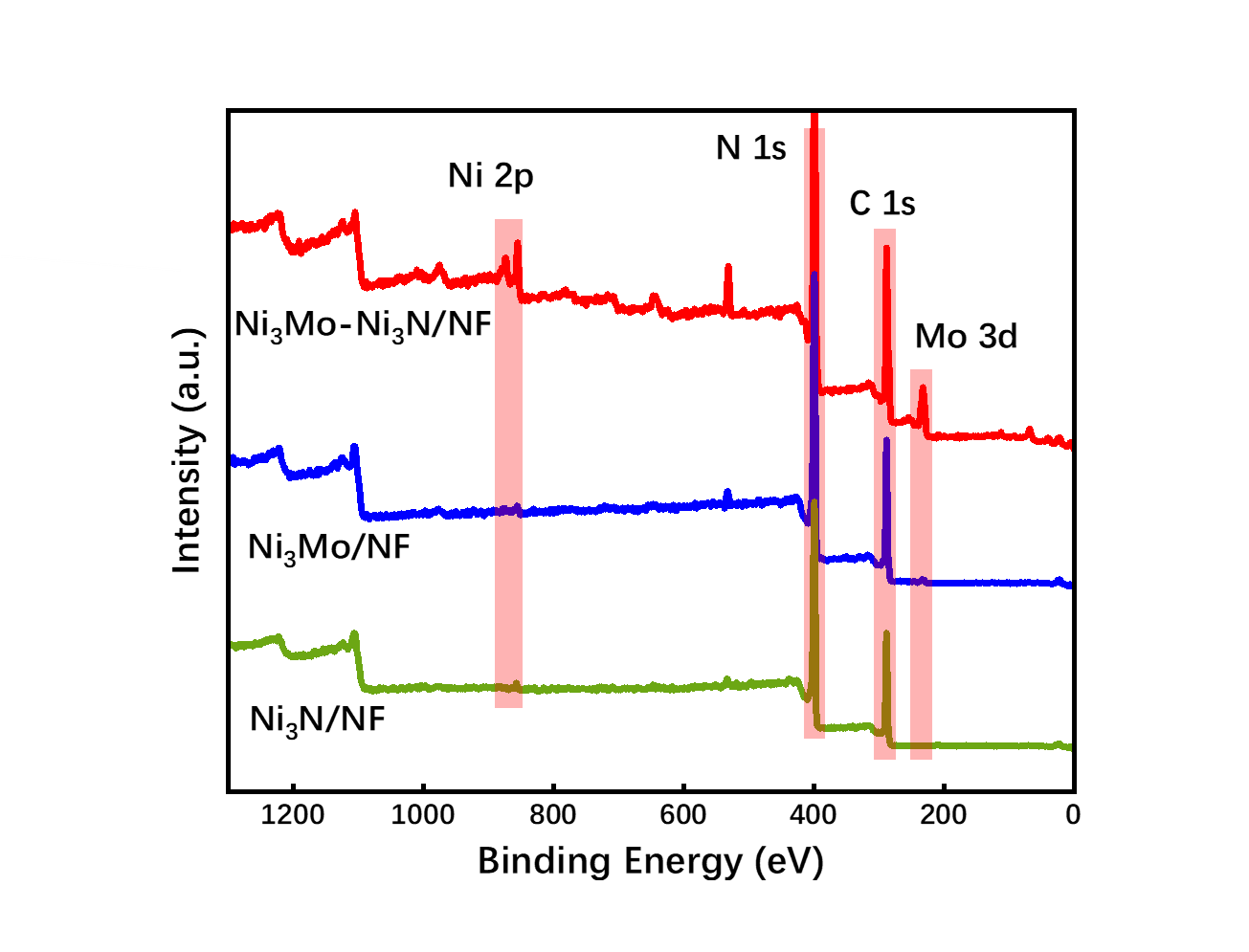
**

**Figure S5.** XPS spectra of Ni_3_Mo-Ni_3_N/NF, Ni_3_Mo/NF, and Ni_3_N/NF.

**
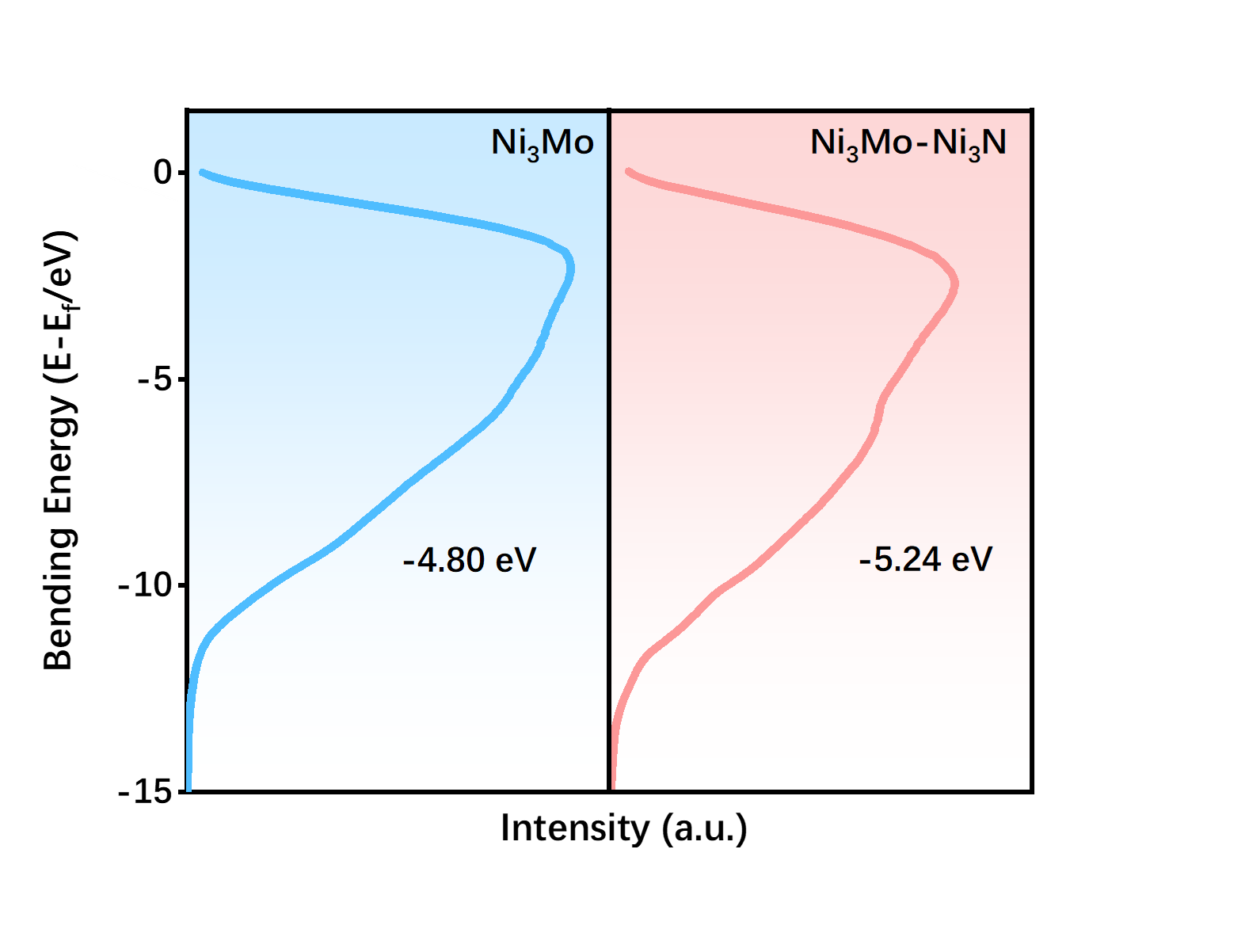
**

**Figure S6.** UPS spectra of Ni_3_Mo/NF and Ni_3_Mo-Ni_3_N/NF.

**
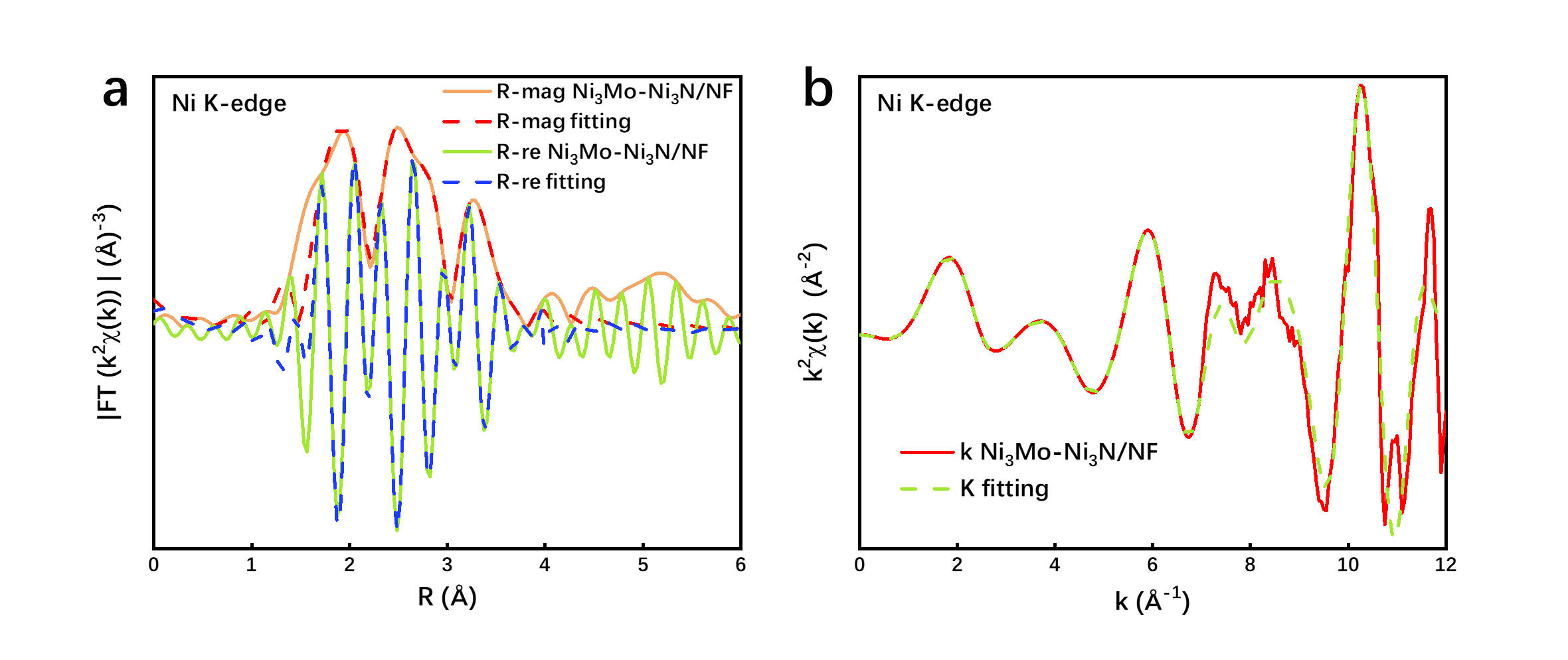
**

**Figure S7.** a) The EXAFS fitting curves about the Ni K-edge of Ni_3_Mo-Ni_3_N/NF in R-space. b) EXAFS oscillation function k^2^𝜒(k) of Ni K-edges.

**
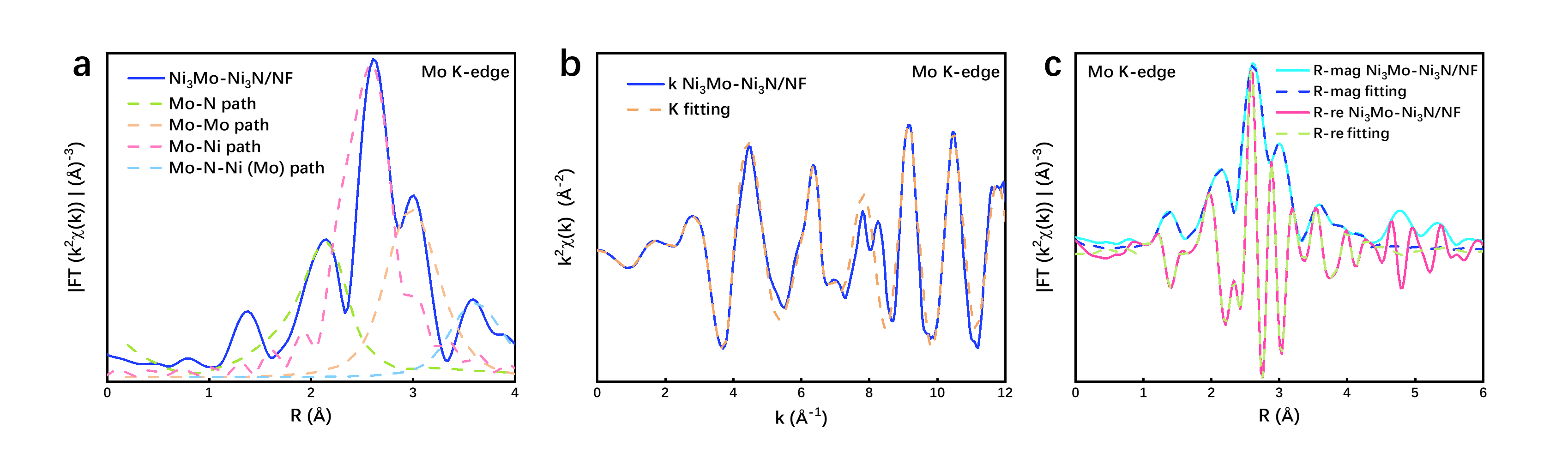
**

**Figure S8.** a) Scattering path of Ni_3_Mo-Ni_3_N/NF Mo K-edge. b) EXAFS oscillation function k^2^𝜒(k) of Mo K-edges. c) The EXAFS fitting curves about the Mo K-edge of Ni_3_Mo-Ni_3_N/NF in R-space.

**
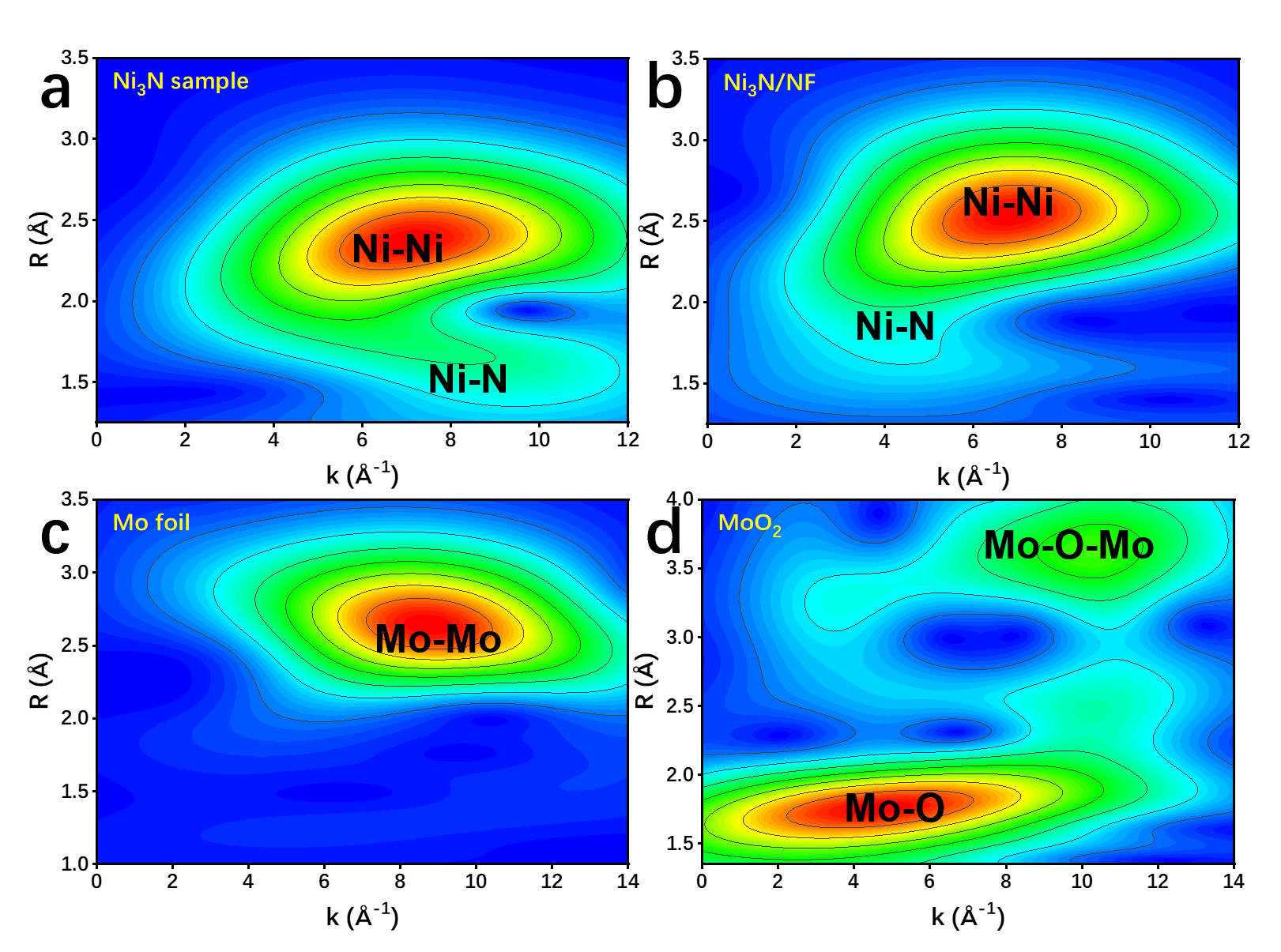
**

**Figure S9.** WT-EXAFS plots of a) Ni_3_N sample and b) Ni_3_N/NF in Ni K-edges. WT-EXAFS plots of c) Mo foil and d) MoO_2_ in Mo K-edge.

**
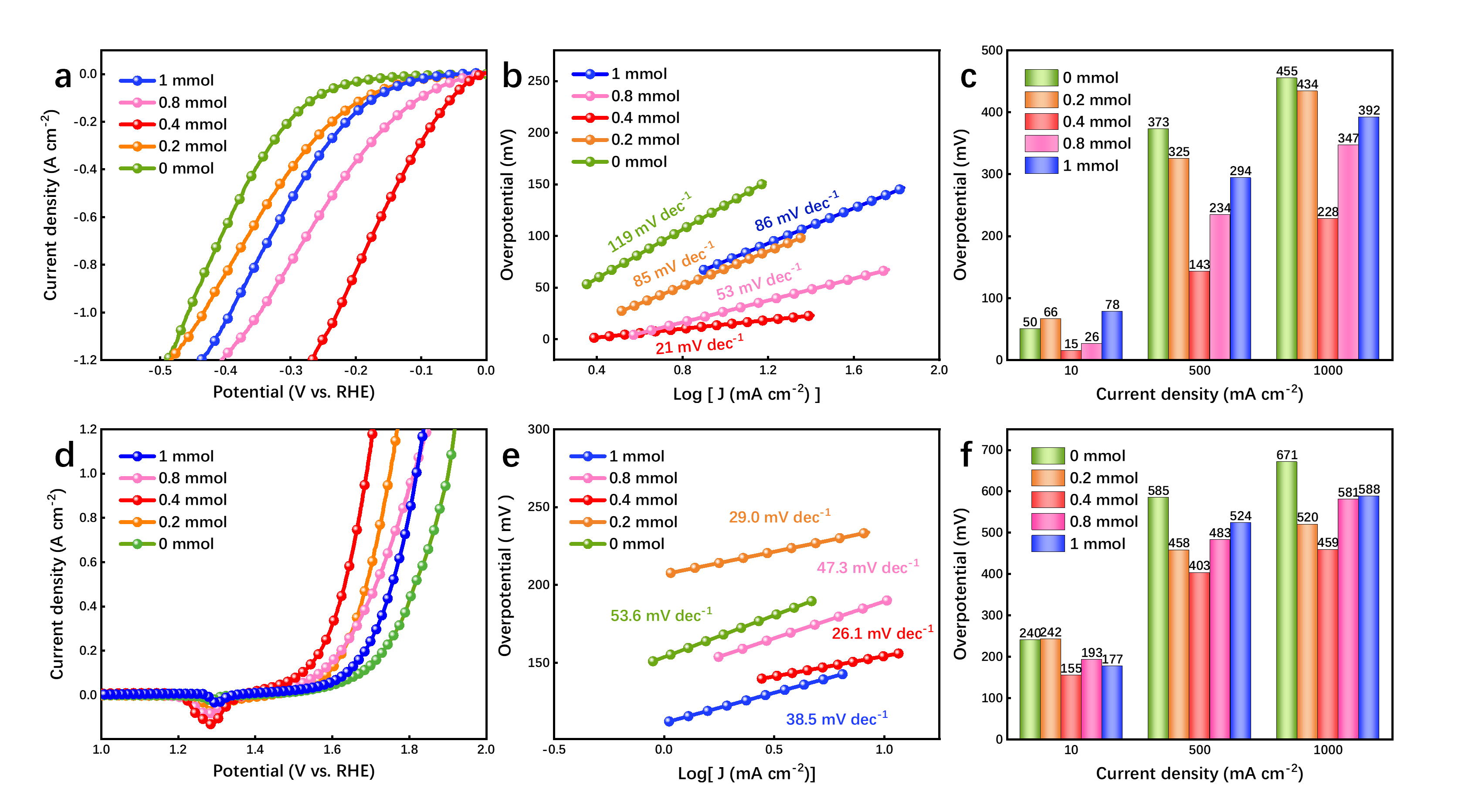
**

**Figure S10.** HER electrochemical characterization with different Mo addition amounts in 1 M KOH electrolyte, a) LSV curves, b) Tafel slopes, and c) HER overpotentials at different current densities. OER electrochemical characterization with different Mo addition amounts in 1 M KOH electrolyte, d) LSV curves, e) Tafel slopes, and f) OER overpotentials at different current densities.

The quantity of Mo was controlled during the synthesis process. Among them, when the amount of Mo added is 0.4 mmol, the corresponding catalyst is Ni_3_Mo-Ni_3_N/NF; for 0 mmol, it corresponds to the Ni_3_N/NF catalyst; and for 1 mmol, it corresponds to the Ni_3_Mo/NF catalyst. When the Mo addition amount is 0.4 mmol, the catalyst exhibits optimal efficiency for both HER and OER, along with the corresponding best Tafel slope.

**
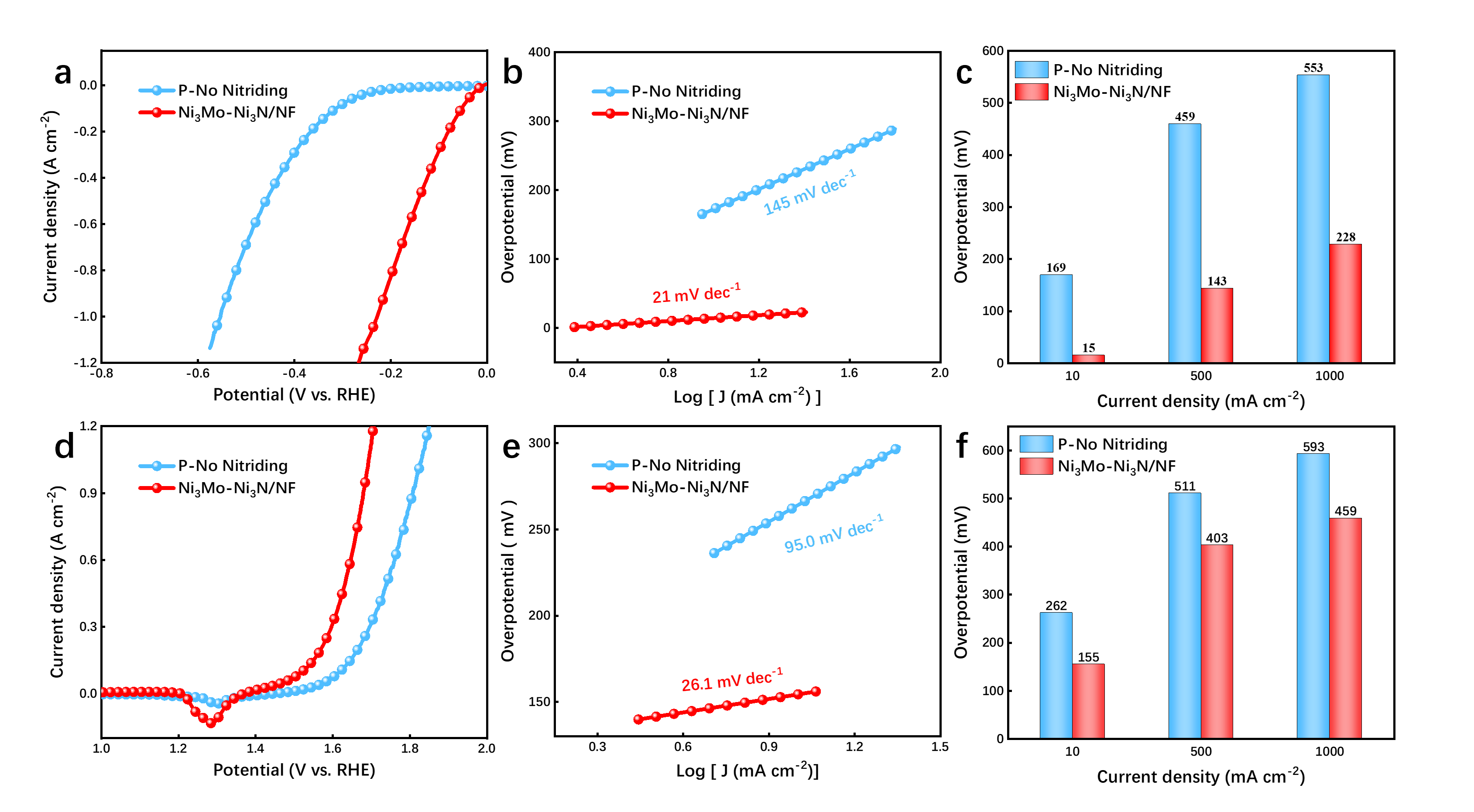
**

**Figure S11.** HER electrochemical characterization before and after nitriding in 1 M KOH electrolyte, a) LSV curves, b) Tafel slopes, and c) HER overpotentials at different current densities. OER electrochemical characterization before and after nitriding in 1 M KOH electrolyte, d) LSV curves, e) Tafel slopes, and f) OER overpotentials at different current densities.

The impact of nitriding on the catalyst's performance was assessed during the synthesis. It is evident that the nitriding process has significantly enhanced both the HER and OER properties of the catalyst.

**
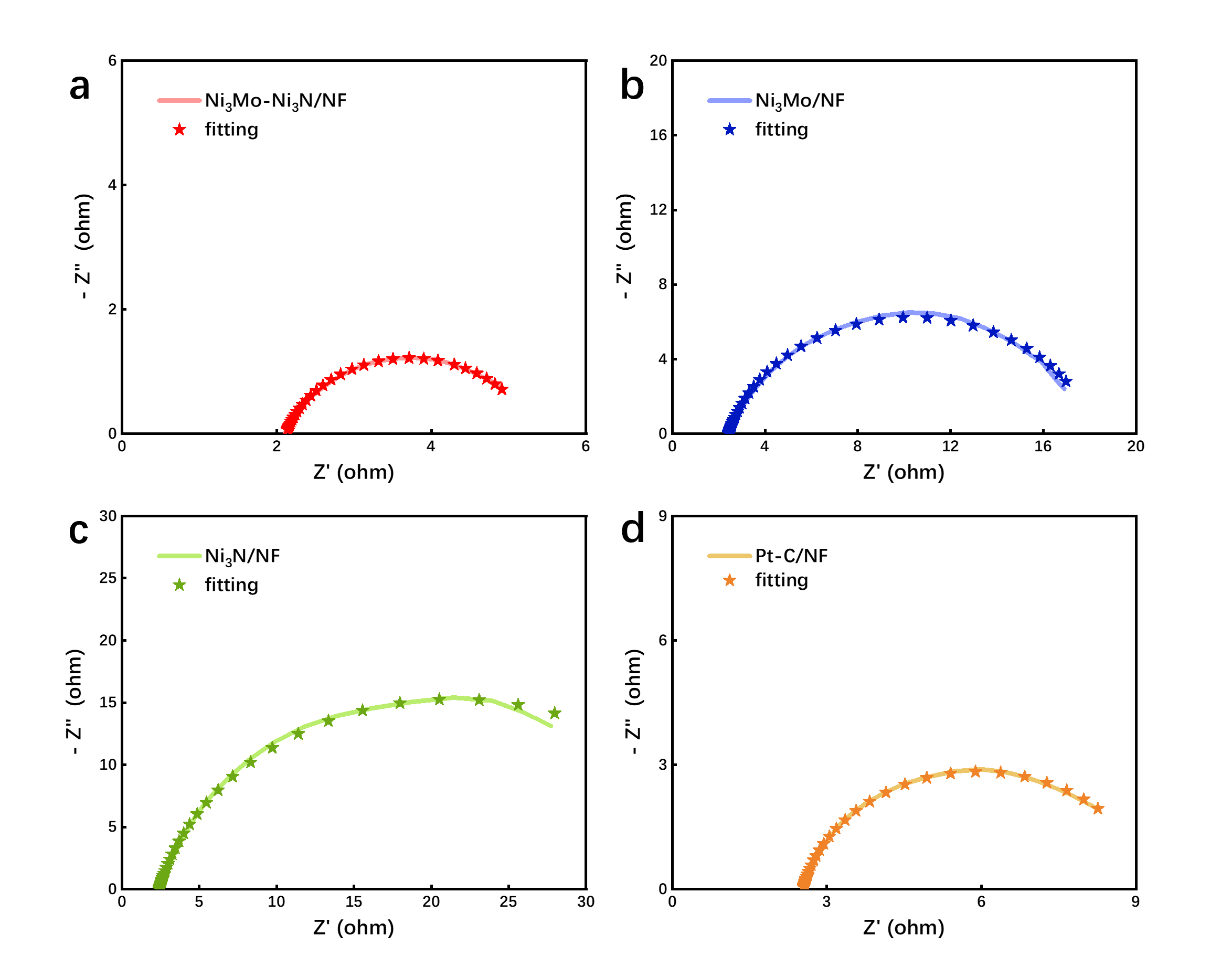
**

**Figure S12.** EIS fitting curves of a) Ni_3_Mo-Ni_3_N/NF, b) Ni_3_Mo/NF, c) Ni_3_N/NF, and d) Pt-C/NF during HER.


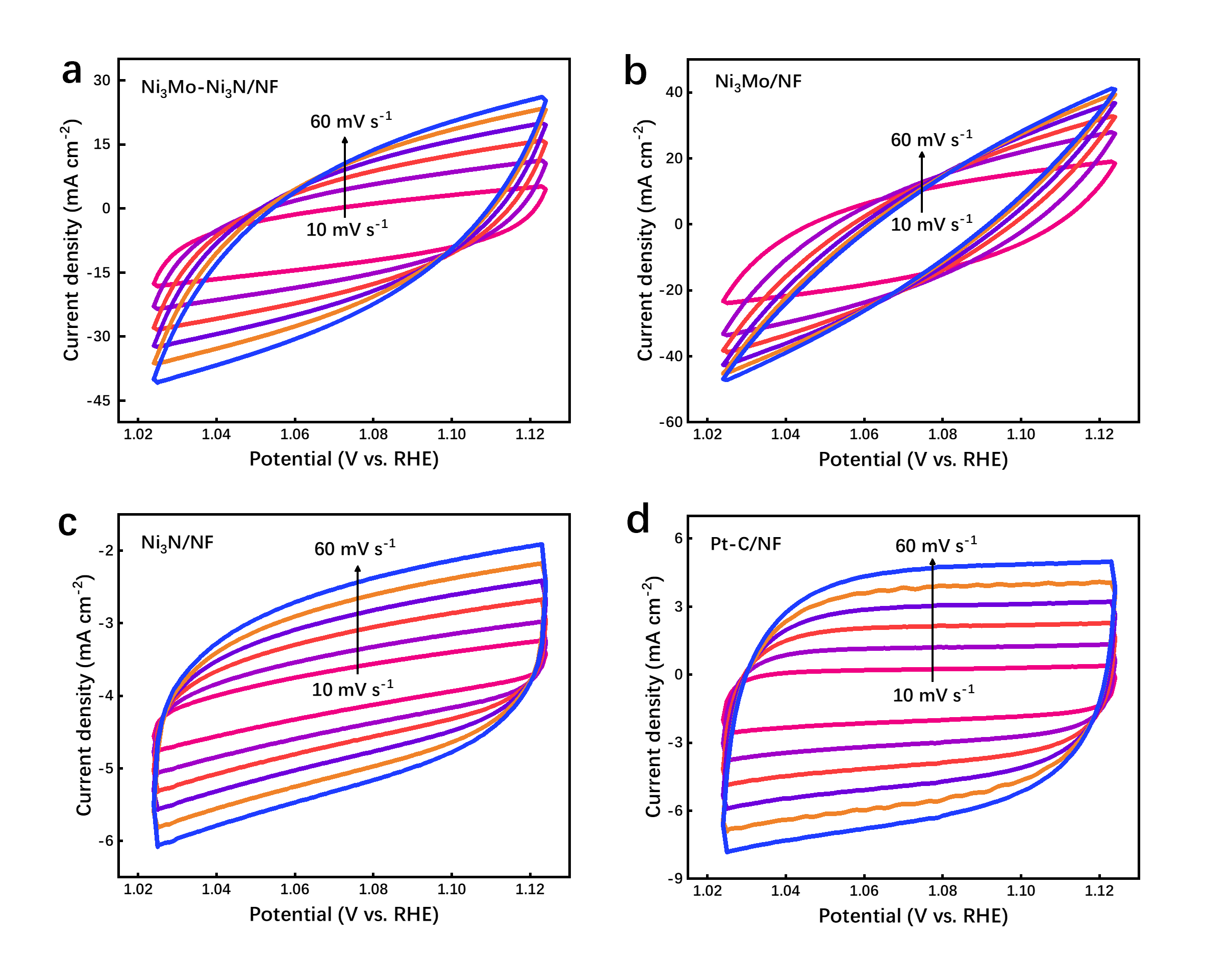


**Figure S13.** CV curves under different scanning speeds of various samples: a) Ni_3_Mo-Ni_3_N/NF, b) Ni_3_Mo/NF, c) Ni_3_N/NF, and d) Pt-C/NF for HER.


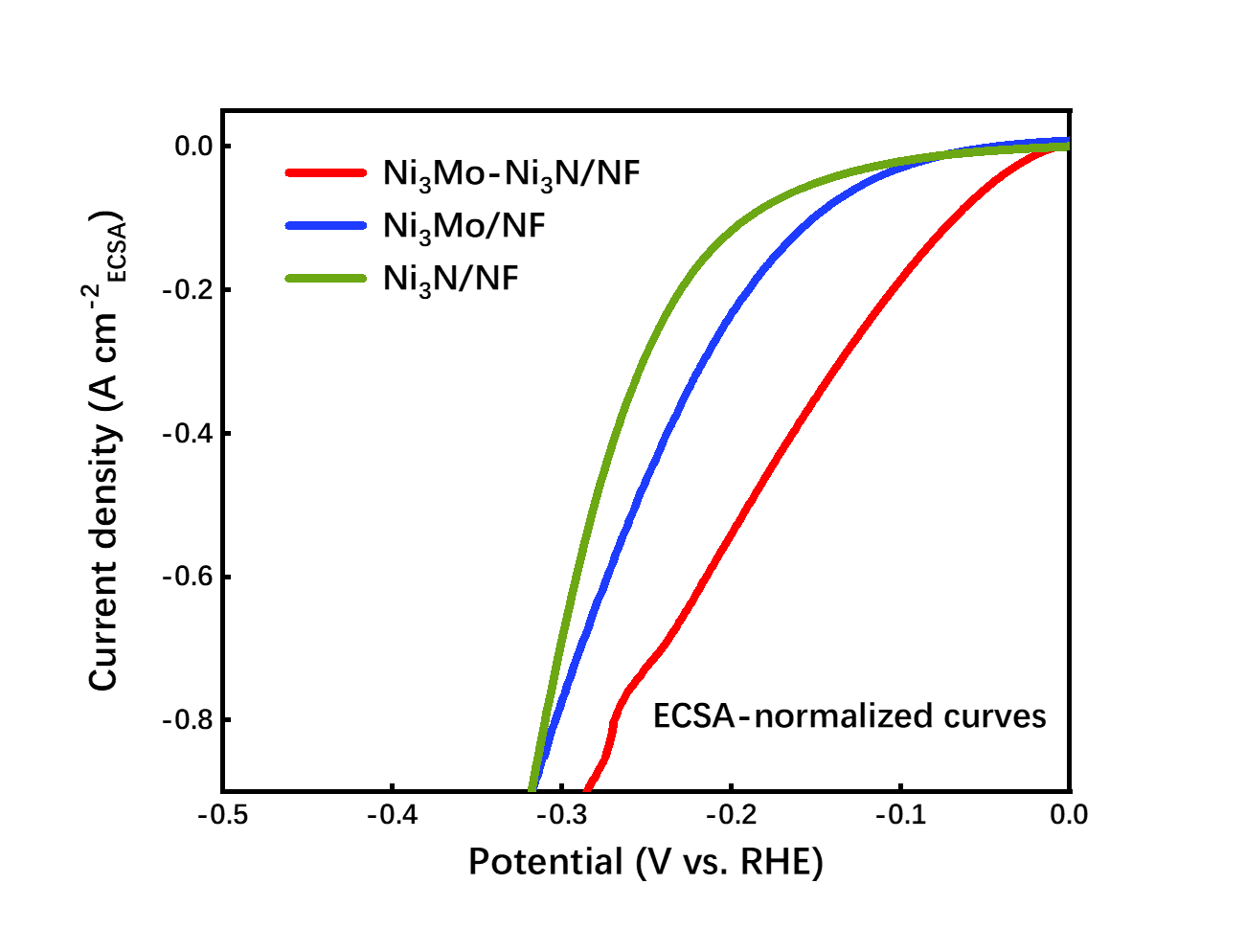


**Figure S14.** ECSA-normalized curves of HER.

ECSA-normalized polarization curves for all catalysts were derived from the ECSA values, which were used to evaluate the intrinsic catalytic activity of the catalysts. In Figure S14, Ni_3_Mo-Ni_3_N/NF exhibits the highest intrinsic catalytic activity for HER, which is consistent with the results of the non-normalized LSV curves. The calculation formula is given as follows:

$$J_{ECSA}=\frac{J_{geo}}{ECSA}$$

**
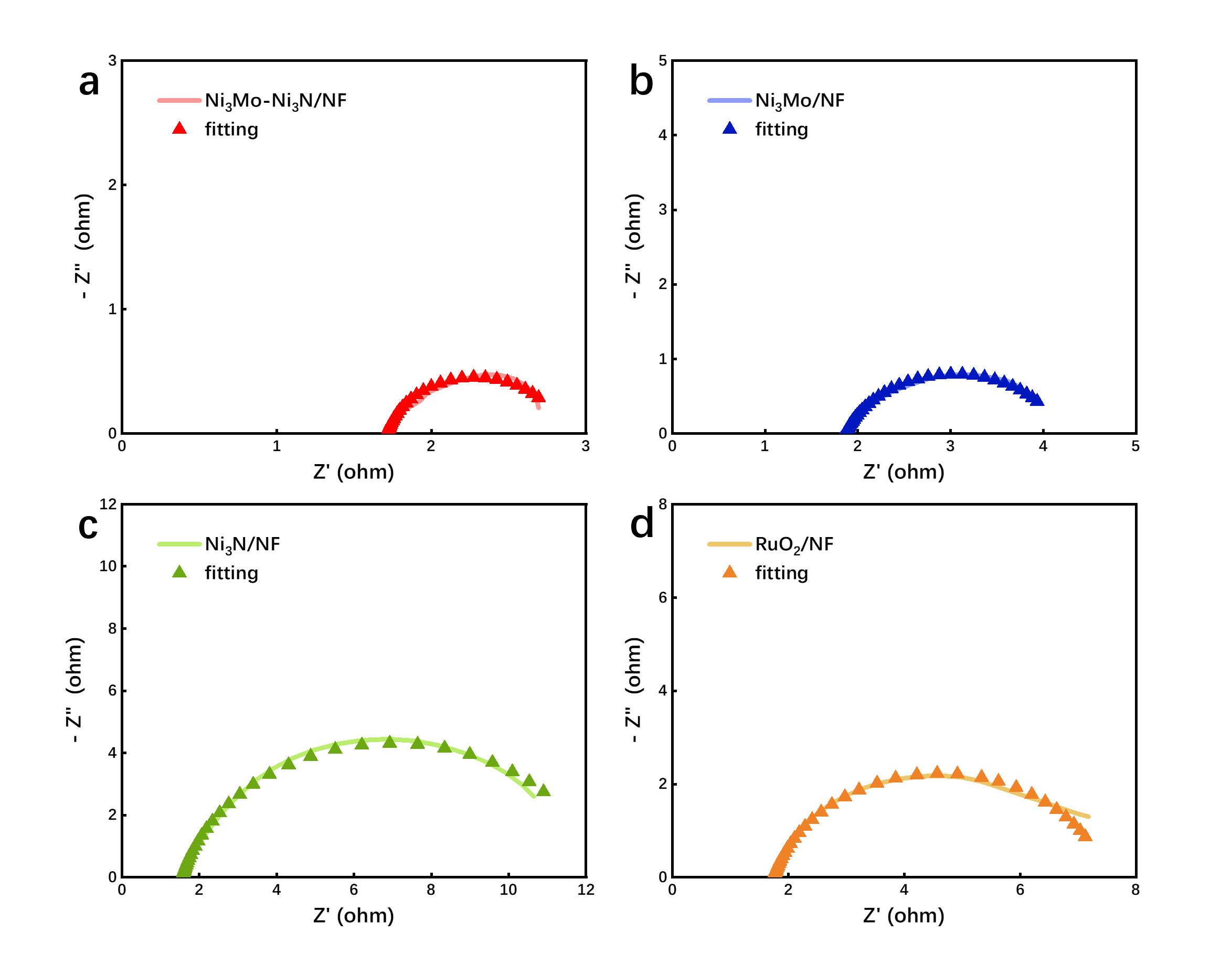
**

**Figure S15.** EIS fitting curves of a) Ni_3_Mo-Ni_3_N/NF, b) Ni_3_Mo/NF, c) Ni_3_N/NF, and d) RuO_2_/NF during OER.

**
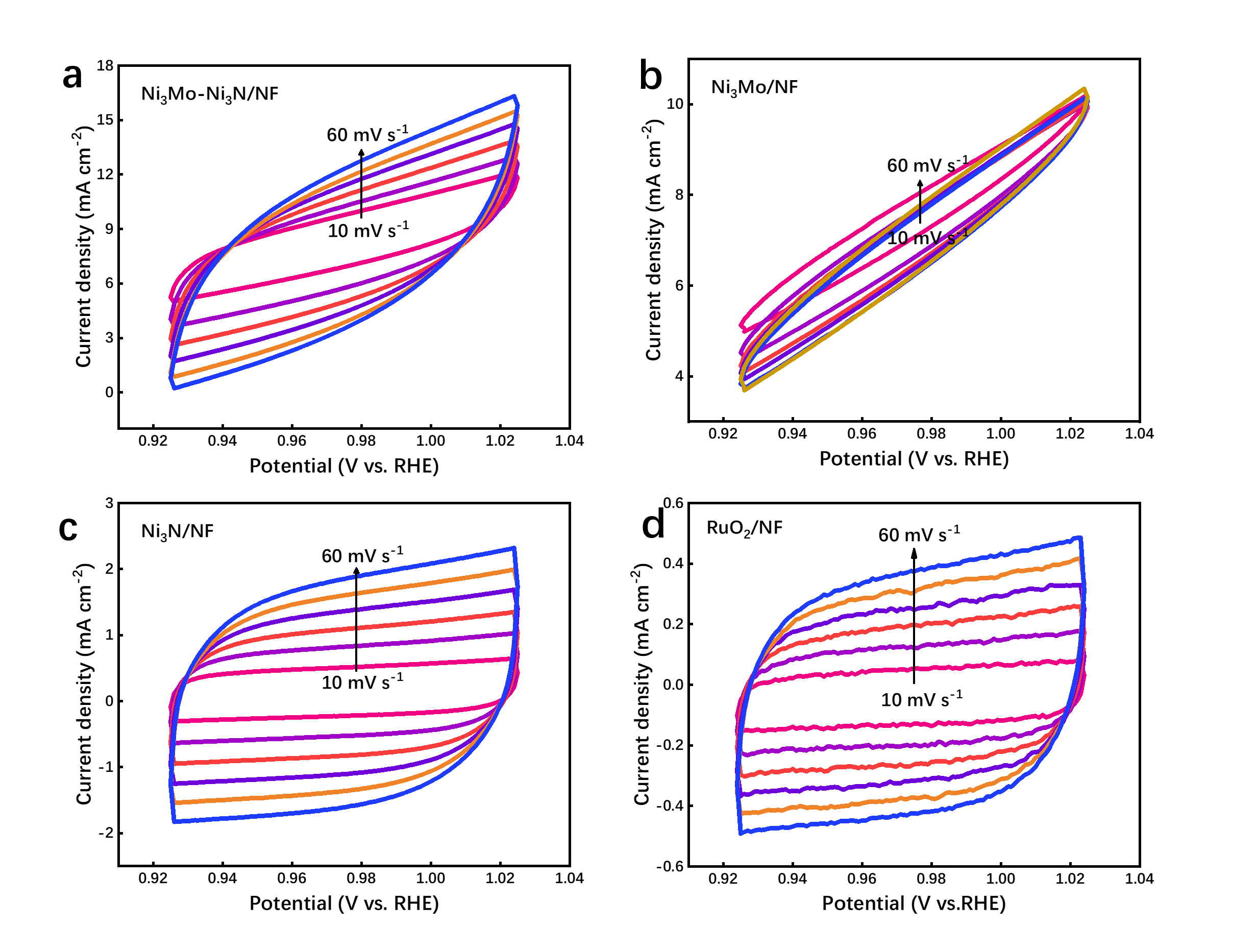
**

**Figure S16.** CV curves under different scanning speeds of various samples: a) Ni_3_Mo-Ni_3_N/NF, b) Ni_3_Mo/NF, c) Ni_3_N/NF, and d) RuO_2_/NF for OER.

**
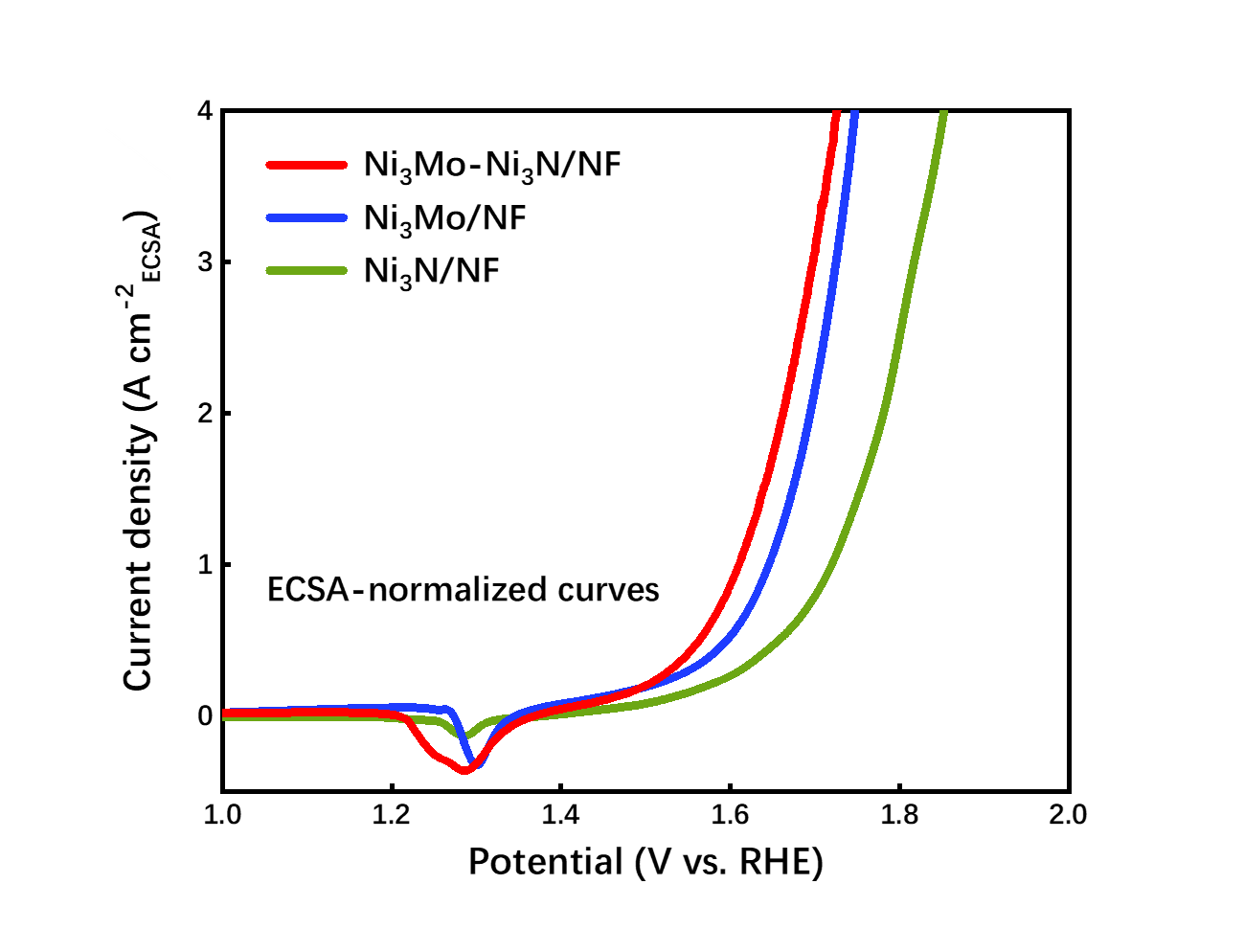
**

**Figure S17.** ECSA-normalized curves of OER.

ECSA-normalized polarization curves for all catalysts were derived from the ECSA values, which were used to evaluate the intrinsic catalytic activity of the catalysts. In Figure S17, Ni_3_Mo-Ni_3_N/NF exhibits the highest intrinsic catalytic activity for OER, which is consistent with the results of the non-normalized LSV curves. The calculation formula is given as follows:

$$J_{ECSA}=\frac{J_{geo}}{ECSA}$$

**Figure S18.** Comparison of recently reported catalysts for overall water splitting.


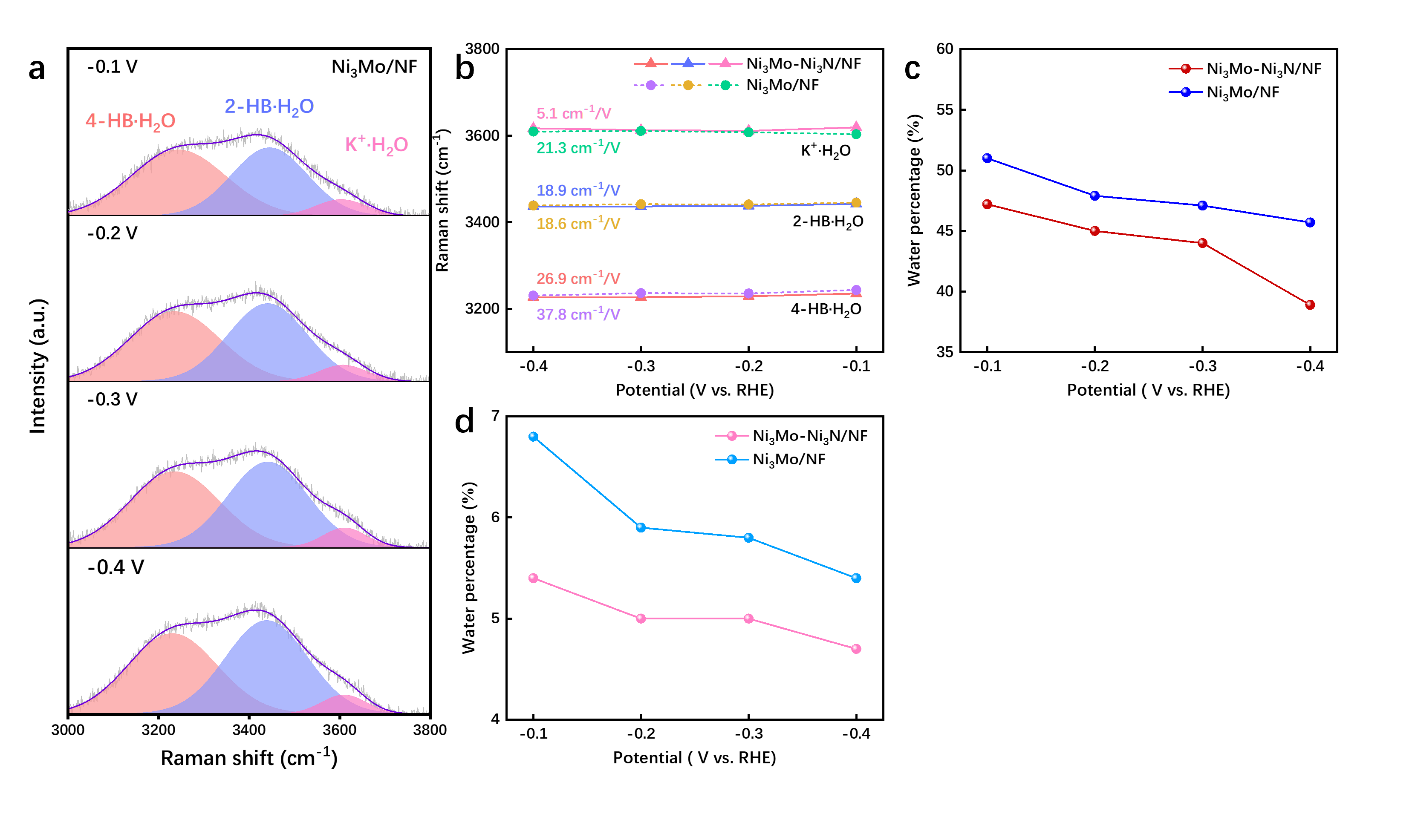


**Figure S19.** a) In situ Raman spectroscopy for the O-H stretching mode from the interfacial water. b) The 𝜈_O-H_ change frequency diagram of interfacial water. Potential-dependence of the ratio of the c) 4-HB·H_2_O and d) K^+^·H_2_O peaks.


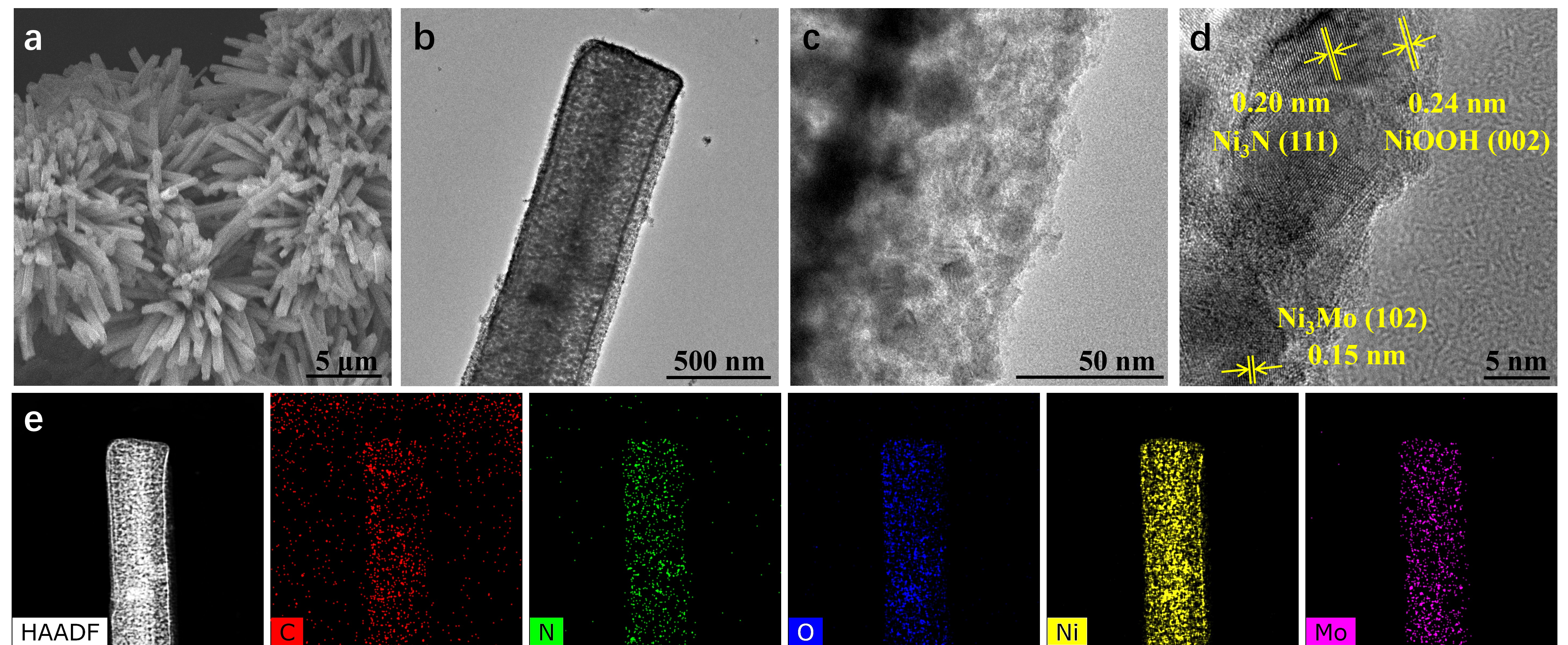


**Figure S20.** a) SEM image, b-c) TEM image, d) HR-TEM image, and e) EDS mapping of Ni_3_Mo-Ni_3_N/NF after OER.

We conducted a systematic morphological and structural analysis on the reconstructed Ni_3_Mo-Ni_3_N/NF catalyst in a 1.0 M KOH electrolyte. As revealed by the SEM and TEM images (Figure S20a,b), the nanorod morphology of the catalyst was well maintained after the OER, showing no significant agglomeration or structural collapse. These observations confirm the excellent structural stability of the catalyst. HR-TEM imaging of the nanorod edges (Figure S20d, from the region in Figure S20c) revealed that the catalyst retained both the (102) crystal plane of Ni_3_Mo and the (111) plane of Ni_3_N. Meanwhile, the newly emerged 0.24 nm lattice stripes at the edge regions belong to the (002) crystal plane of NiOOH. This confirms that during the OER, the nanorods underwent only a limited surface reconstruction to form NiOOH, rather than a drastic bulk phase transformation. In addition, the acquired energy dispersive spectroscopy (EDS) mapping shows that the elements C, N, O, Ni, and Mo in Ni_3_Mo-Ni_3_N/NF are uniformly distributed across the nanorods (Figure S20e).


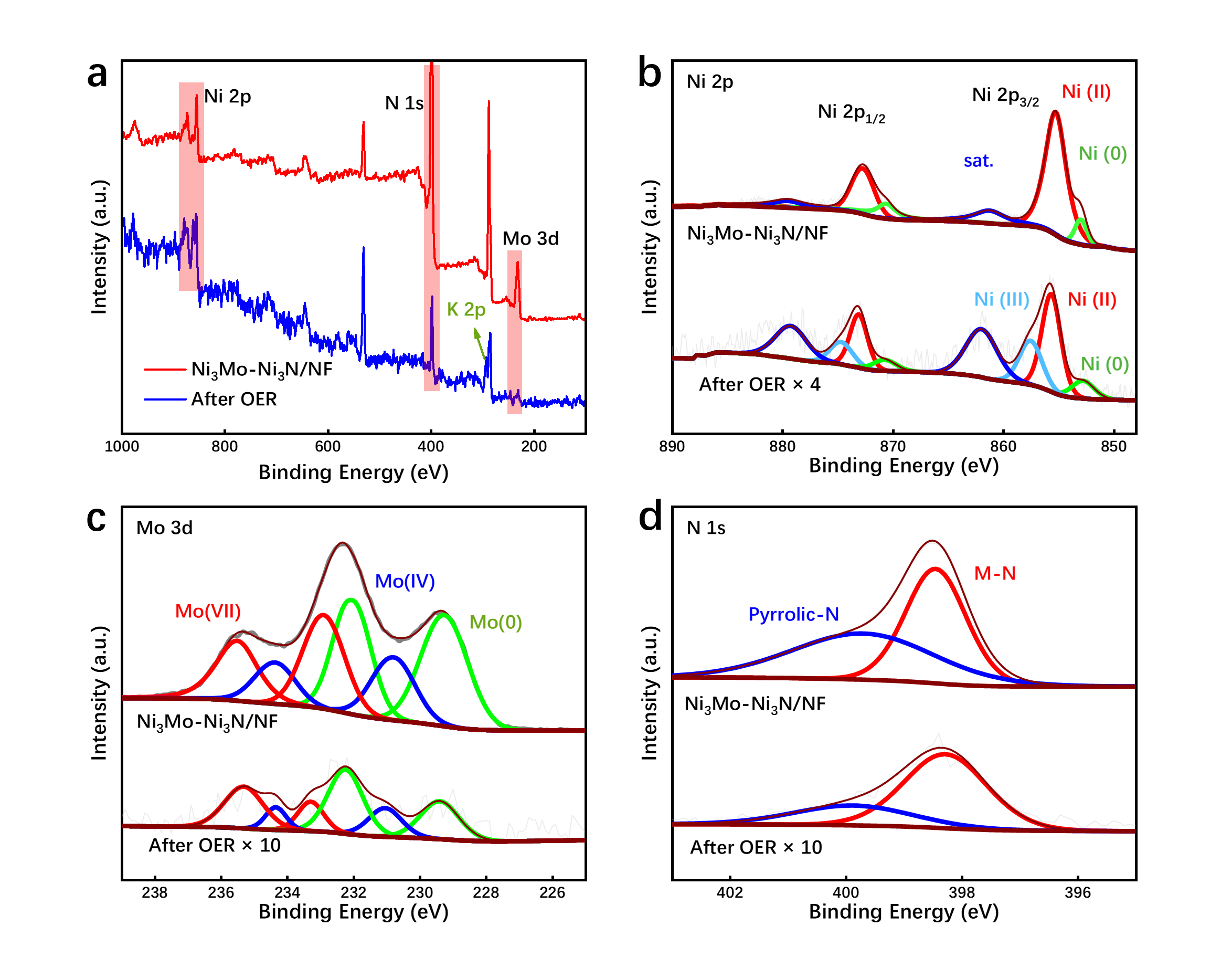


**Figure S21.** a) XPS spectra of Ni_3_Mo-Ni_3_N/NF and Ni_3_Mo-Ni_3_N/NF after OER for b) Ni 2*p*, c) Mo 3*d*, and d) N 1*s*.

We tested the XPS spectra of the catalyst and conducted structural analysis. Figure S21a shows the comparison of full elements of the sample before and after reconstruction, revealing that elements C, N, O, Ni, and Mo remain present after OER. The Ni *2p* XPS spectra (Figure S21b) show a characteristic peak at 857.5 eV after OER, corresponding to Ni^3+^. This further confirms the formation of NiOOH species through surface reconstruction. In the XPS spectra of Mo 3*d* (Figure S21c) and N 1*s* (Figure S21d), there are no significant differences were observed before and after reconstruction.


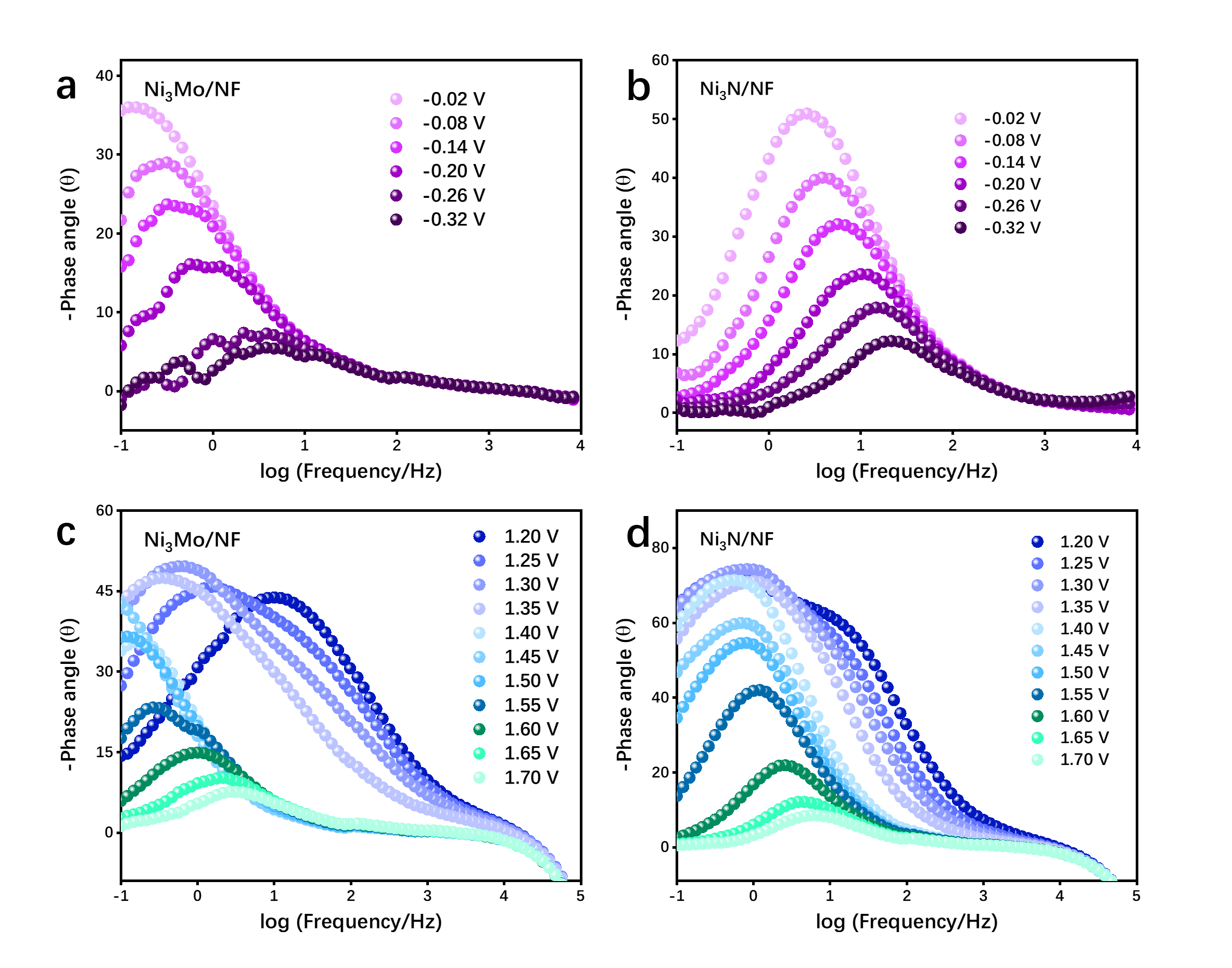


**Figure S22.** a-b) HER and c-d) OER EIS Bode plots of Ni_3_Mo/NF and Ni_3_N/NF.


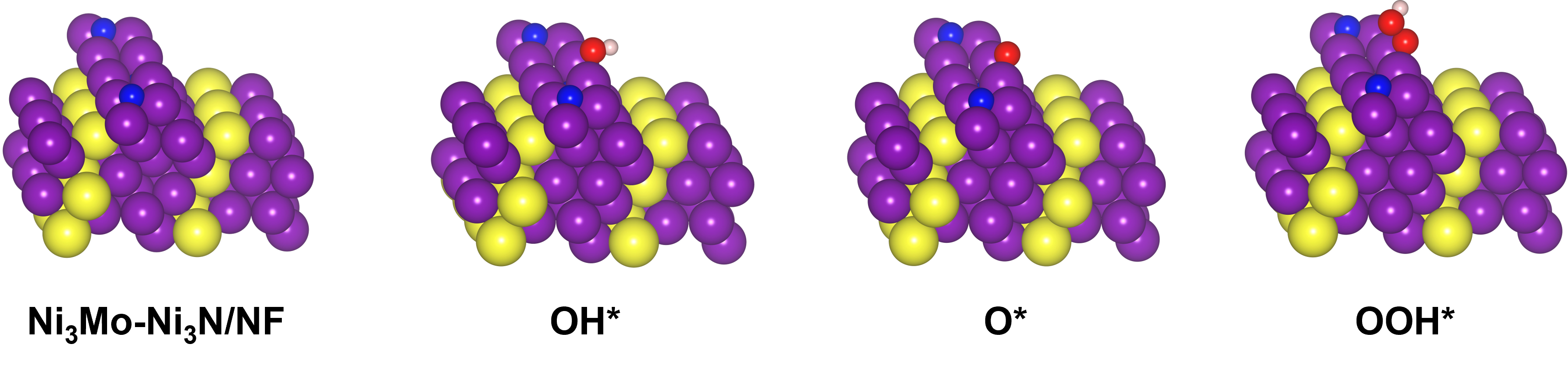


**Figure S23.** The atomic models and OER overreaction states of Ni_3_Mo-Ni_3_N/NF.


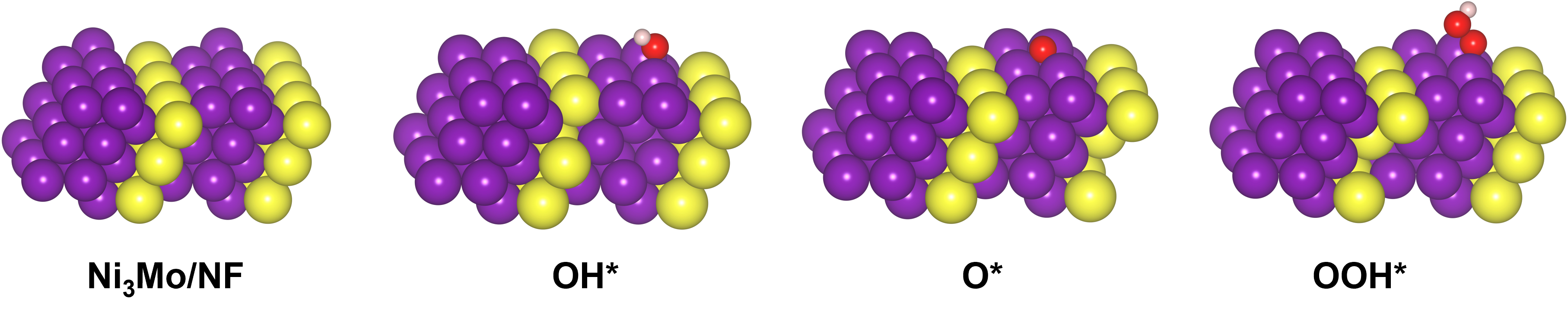


**Figure S24.** The atomic models and OER overreaction states of Ni_3_Mo/NF.


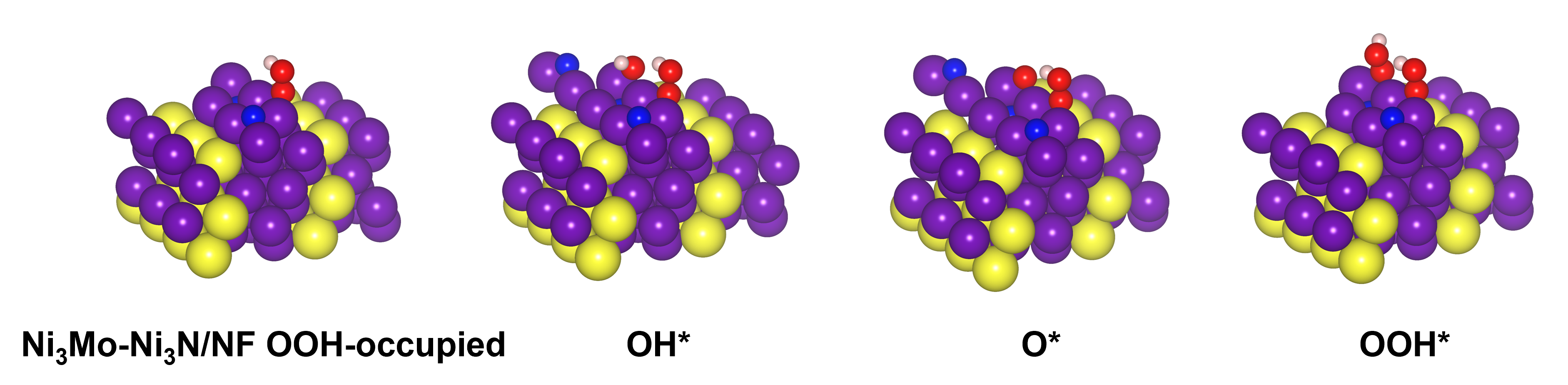


**Figure S25.** The atomic models and OER overreaction states of Ni_3_Mo-Ni_3_N/NF with -OOH occupied.

**Table S1.** EXAFS fitting results for the local structure parameters around Ni of Ni_3_Mo-Ni_3_N/NF.

|  | **Reduced Chi-square (χ_ν_^2^)** | **R-factor（%）** | **amp/ S_0_^2^** | **N_(Ni-N path)_** | **R_(Ni-N path)_ (Å)** | **σ^2^_(Ni-N path)_ (10^-3^ Å^2^)** | **ΔE_0_ (eV)** |
| --- | --- | --- | --- | --- | --- | --- | --- |
| **Ni**  Ni_3_Mo-Ni_3_N/  NF | 526.77 | 0.0411 | 0.85±0.13 | 5 | 1.891±0.076 | 2.2±1.1 | 2.25±1.04 |
|  |  |  | **amp/ S_0_^2^** | **N_(Ni-Ni path)_** | **R_(Ni-Ni path)_ (Å)** | **σ^2^_(Ni-Ni path)_ (10^-3^ Å^2^)** | **ΔE_0_ (eV)** |
|  |  |  | 0.91±0.14 | 2 | 2.471±0.028 | 1.9±0.7 | 2.45±0.72 |
|  |  |  | **amp/ S_0_^2^** | **N_(Ni-Mo path)_** | **R_(Ni-Mo path)_ (Å)** | **σ^2^_(Ni-Mo path)_ (10^-3^ Å^2^)** | **ΔE_0_ (eV)** |
|  |  |  | 0.94±0.12 | 2 | 2.728± 0.092 | 1.8±0.9 | 2.43±0.98 |
|  |  |  | **amp/ S_0_^2^** | **N_(Ni-N-Ni (Mo) path)_** | **R_(Ni-N-Ni (Mo) path)_**  **(Å)** | **σ^2^_(Ni-N-Ni (Mo) path)_**  **(10^-3^ Å^2^)** | **ΔE_0_ (eV)** |
|  |  |  | 0.93±0.15 | 2 | 3.263±0.062 | 2.2±1.2 | 2.21±1.06 |

**Table S2.** EXAFS fitting results for the local structure parameters around Mo of Ni_3_Mo-Ni_3_N/NF.

|  | **Reduced Chi-square (χ_ν_^2^)** | **R-factor（%）** | **amp/ S_0_^2^** | **N_(Mo-N path)_** | **R_(Mo-N path)_ (Å)** | **σ^2^_(Mo-N path)_ (10^-3^ Å^2^)** | **ΔE_0_ (eV)** |
| --- | --- | --- | --- | --- | --- | --- | --- |
| **Mo** Ni_3_Mo-Ni_3_N/  NF | 664.88 | 0.0399 | 0.95±0.11 | 3 | 1.998±0.074 | 2.2±1.1 | 2.24±1.12 |
|  |  |  | **amp/ S_0_^2^** | **N_(Mo-Ni path)_** | **R_(Mo-Ni path)_ (Å)** | **σ^2^_(Mo-Ni path)_ (10^-3^ Å^2^)** | **ΔE_0_ (eV)** |
|  |  |  | 0.93±0.14 | 2 | 2.649±0.092 | 1.9±0.7 | 2.18±0.44 |
|  |  |  | **amp/ S_0_^2^** | **N_(Mo-Mo path)_** | **R_(Mo-Mo path)_ (Å)** | **σ^2^_(Mo-Mo path)_ (10^-3^ Å^2^)** | **ΔE_0_ (eV)** |
|  |  |  | 0.95±0.15 | 2 | 2.814±0.075 | 2.0±1.1 | 2.05±0.76 |
|  |  |  | **amp/ S_0_^2^** | **N****_(Mo-N-Ni (Mo) path)_** | **R_(Mo-N-Ni (Mo) path)_**  **(Å)** | **σ^2^_(Mo-N-Ni (Mo) path)_**  **(10^-3^ Å^2^)** | **ΔE_0_ (eV)** |
|  |  |  | 0.96±0.11 | 2 | 3.419±0.086 | 2.4±1.2 | 2.79±0.81 |

**Table S3.** EIS fitting result of Ni_3_Mo-Ni_3_N/NF in the HER process.

| **Element** | **Value** | **Error%** |
| --- | --- | --- |
| **R_Ω_** | 2.119 | 0.211 |
| **CPE-T** | 0.001 | 3.314 |
| **CPE-P** | 0.827 | 0.536 |
| **R_ct_** | 3.204 | 0.717 |

**Table S4.** EIS fitting result of Ni_3_Mo/NF in the HER process.

| **Element** | **Value** | **Error%** |
| --- | --- | --- |
| **R_Ω_** | 2.428 | 0.377 |
| **CPE-T** | 0.010 | 1.618 |
| **CPE-P** | 0.850 | 0.515 |
| **R_ct_** | 15.830 | 0.909 |

**Table S5.** EIS fitting result of Ni_3_N/NF in the HER process.

| **Element** | **Value** | **Error%** |
| --- | --- | --- |
| **R_Ω_** | 2.400 | 0.452 |
| **CPE-T** | 0.009 | 1.658 |
| **CPE-P** | 0.862 | 0.561 |
| **R_ct_** | 38.040 | 1.919 |

**Table S6.** EIS fitting result of Pt-C/NF in the HER process.

| **Element** | **Value** | **Error%** |
| --- | --- | --- |
| **R_Ω_** | 2.539 | 0.239 |
| **CPE-T** | 0.005 | 1.984 |
| **CPE-P** | 0.884 | 0.467 |
| **R_ct_** | 6.805 | 0.806 |

**Table S7.** The mass of the catalysts loaded on a 0.5 × 0.5 cm^2^ Ni foam.

| **Catalysts** | **Mass on 0.5 × 0.5 cm^2^ Ni foam (mg)** |
| --- | --- |
| Ni_3_Mo-Ni_3_N/NF | 1.87 |
| Ni_3_Mo/NF | 1.73 |
| Ni_3_N/NF | 0.95 |

**Table S8.** Comparison of HER electrocatalytic activity in 1 M KOH of the catalysts reported in this work.

| **Catalysts** | **ECSA**  **[cm^2^]** | **ECSA**  **[m^2^ g^-1^]** | **Current density at**  **300 mV [mA cm^-2^]** | **TOF**  **[s^-1^]** | **Mass activity at 300 mV [A g^-1^]** |
| --- | --- | --- | --- | --- | --- |
| Ni_3_Mo-Ni_3_N/NF | 764 | 41 | 1436 | 1.46 | 191.82 |
| Ni_3_Mo/NF | 343 | 20 | 530 | 1.22 | 76.79 |
| Ni_3_N/NF | 138 | 15 | 191 | 1.08 | 50.14 |

The ECSA of each catalyst for the HER were calculated based on the double-layer capacitance. Meanwhile, the ECSA per unit mass was computed using the loading density of each catalyst on the nickel foam surface. It was concluded that the Ni_3_Mo-Ni_3_N/NF catalyst exhibits the largest ECSA for HER. The calculation formulas are provided as follows:

$$ECSA=\frac{C_{dl}}{C_{s}}$$

where C_dl_ enotes the double-layer capacitance with the unit of mF. C_s_ represents the specific capacitance, which is 0.04 mF cm^-2^ in 1 M KOH electrolyte.

TOF is a key parameter used to quantify the number of reaction events occurring at each active site per unit time. We calculated the TOF values of different catalysts based on the current densities measured at an overpotential of 300 mV. The Ni_3_Mo-Ni_3_N/NF catalyst exhibits higher TOF values for HER than the other catalysts, demonstrating its superior catalytic performance. The calculation formula is given as follows:

$$TOF=\frac{jS}{2Fn}$$

where j represents the current density corresponding to a certain voltage of HER. S (0.25 cm^2^) is the reaction area of the electrode. z is the number of electron transfers during the reaction process. F is the Faraday constant (96485.3 C·mol^-1^). n is the total number of moles of active metal atoms on the electrode surface for the reaction area.

Subsequently, we further evaluated the catalytic activity via mass activity, which was calculated based on the measured current density j (mA·cm^-2^) at an overpotential of 300 mV, as well as the catalyst loading density m (mg·cm^-2^). The Ni_3_Mo-Ni_3_N/NF catalyst exhibited the highest mass activity for HER, which further confirms its superior catalytic performance toward both reactions. The calculation formula is given as follows:

$$Mass activity=\frac{j}{m}$$

**Table S9.** Comparison of recently reported heterojunction catalysts for the HER at 10 mA cm^−2^ in 1 M KOH.

| Catalyst | Overpotential | Stability | Reference |
| --- | --- | --- | --- |
| Ni_3_Mo-Ni_3_N/NF | 15 mV | 1000 h | This work |
| NiMo/Mo_2_N/NC | 5.8 mV | 1000 h | *Appl. Catal. B Environ.*, **2023,** *343*, 123362 |
| Mo_2_N/Ni_3_Mo_3_N/NF | 9.4 mV | 100 h | *Adv. Funct. Mater*., **2024**, *34*, 2306061 |
| Os/OsSe_2_ | 23 mV | 30 h | *Angew. Chem. Int. Ed*., **2022**, *61*, e202208642 |
| Co/MoN/NC | 29 mV | 90 h | *Chin. J. Struct. Chem*., **2022**, *41*, 2207040-2207045 |
| MoS_2_/CoS_2_ | 46 mV | 0 | *Phys. Chem. Chem. Phys.*, **2023**, *25*, 13966 |
| Co_2_P/MoNiP/NF | 46 mV | 100 h | *Chem. Eng. J.*, **2023**, *477*, 147092 |
| Co_2_P/Co_4_N | 53 mV | 50 h | *Chem. Eng. J.*, **2023**, *454*, 140230 |
| Co/NiCoP/CC | 54 mV | 30 h | *J. Alloys Compd.*, **2022**, *929*, 167229 |
| MoN/NiFe-LDH/CC | 62 mV | 20 h | *Small*, **2023**, *19*, 2303932 |
| NiSe_2_/MoSe_2_/CC | 79 mV | 48 h | *Appl. Catal. B Environ.*, **2024**, *341*, 123312 |
| MoO_2_/WO_2_@N,P-CNFs | 95 mV | 40 h | *Rare Met.*, **2024**, *43*, 489-499 |
| MoC/Mo_2_C | 98 mV | 1000 h | *Nat. Commun.*, **2021**, *12*, 6776 |
| NiO/CeO_2_ | 99 mV | 20 h | *J. Colloid Interface Sci.*, **2023**, *643*, 282-291 |
| MoO_2_/E-MoS_2_ | 99 mV | 130 h | *Appl. Catal. B Environ.*, **2024,** *343*, 123534 |
| SnS_2_/SnO_2_/NF | 108 mV | 30 h | *Appl. Catal. B Environ.*, **2021,** *292*, 120200 |
| Co_6_Mo_6_C_2_/Co/NC | 114 mV | 60 h | *Appl. Catal. B Environ.*, **2023,** *334*, 122830 |
| Co_2_P/CoP | 121 mV | 25 h | *Nano Res*., **2023**, *16*, 12830-12839 |
| MoNi/NiMoO_x_/NF | 139 mV | 100 h | *Adv. Funct. Mater.*, **2023**, *33*, 2307109 |
| MoSe_2_/CoSe_2_/NF | 180 mV | 18 h | *J. Colloid Interface Sci.*, **2024**, *658*, 32-42 |
| Cu_2-x_Se/MoSe_2_ | 198 mV | 10 h | *Small*, **2023**, 2308650 |

Note: NF: Ni foam; CC: carbon cloth; NC: nanocube.

**Table S10.** EIS fitting result of Ni_3_Mo-Ni_3_N/NF in the OER process.

| **Element** | **Value** | **Error%** |
| --- | --- | --- |
| **R_Ω_** | 1.715 | 0.329 |
| **CPE-T** | 0.425 | 2.488 |
| **CPE-P** | 0.854 | 1.770 |
| **R_ct_** | 1.143 | 2.346 |

**Table S11.** EIS fitting result of Ni_3_Mo/NF in the OER process.

| **Element** | **Value** | **Error%** |
| --- | --- | --- |
| **R_Ω_** | 1.860 | 0.249 |
| **CPE-T** | 0.148 | 1.958 |
| **CPE-P** | 0.756 | 1.044 |
| **R_ct_** | 2.363 | 1.287 |

**Table S12.** EIS fitting result of Ni_3_N/NF in the OER process.

| **Element** | **Value** | **Error%** |
| --- | --- | --- |
| **R_Ω_** | 1.553 | 0.342 |
| **CPE-T** | 0.044 | 1.182 |
| **CPE-P** | 0.851 | 0.525 |
| **R_ct_** | 10.920 | 1.099 |

**Table S13.** EIS fitting result of RuO_2_/NF in the OER process.

| **Element** | **Value** | **Error%** |
| --- | --- | --- |
| **R_Ω_** | 1.748 | 0.515 |
| **CPE-T** | 0.001 | 4.674 |
| **CPE-P** | 0.838 | 0.735 |
| **R_ct_** | 5.760 | 0.979 |

**Table S14.** Comparison of OER electrocatalytic activity in 1 M KOH of the catalysts reported in this work.

| **Catalysts** | **ECSA**  **[cm^2^]** | **ECSA**  **[m^2^ g^-1^]** | **Current density at**  **1.7 V [mA cm^-2^]** | **TOF**  **[s^-1^]** | **Mass activity at η=1.7 V [A g^-1^]** |
| --- | --- | --- | --- | --- | --- |
| Ni_3_Mo-Ni_3_N/NF | 366 | 20 | 1120 | 1.19 | 149.56 |
| Ni_3_Mo/NF | 114 | 7 | 246 | 0.84 | 35.66 |
| Ni_3_N/NF | 170 | 18 | 135 | 0.31 | 35.45 |

The ECSA of each catalyst for the OER were calculated based on the double-layer capacitance. Meanwhile, the ECSA per unit mass was computed using the loading density of each catalyst on the nickel foam surface. It was concluded that the Ni_3_Mo-Ni_3_N/NF catalyst exhibits the largest ECSA for OER. The calculation formulas are provided as follows:

$$ECSA=\frac{C_{dl}}{C_{s}}$$

where C_dl_ enotes the double-layer capacitance with the unit of mF. C_s_ represents the specific capacitance, which is 0.04 mF cm^-2^ in 1 M KOH electrolyte.

TOF is a key parameter used to quantify the number of reaction events occurring at each active site per unit time. We calculated the TOF values of different catalysts based on the current densities measured at an applied voltage of 1.7 V. The Ni_3_Mo-Ni_3_N/NF catalyst exhibits higher TOF values for OER than the other catalysts, demonstrating its superior catalytic performance. The calculation formula is given as follows:

$$TOF=\frac{jS}{4Fn}$$

where j represents the current density corresponding to a certain voltage of OER. S (0.25 cm^2^) is the reaction area of the electrode. z is the number of electron transfers during the reaction process. F is the Faraday constant (96485.3 C·mol^-1^). n is the total number of moles of active metal atoms on the electrode surface for the reaction area.

Subsequently, we further evaluated the catalytic activity via mass activity, which was calculated based on the measured current density j (mA·cm^-2^) at an applied voltage of 1.7 V, as well as the catalyst loading density m (mg·cm^-2^). The Ni_3_Mo-Ni_3_N/NF catalyst exhibited the highest mass activity for OER, which further confirms its superior catalytic performance toward both reactions. The calculation formula is given as follows:

$$Mass activity=\frac{j}{m}$$

**Table S15.** Comparison of recently reported heterojunction catalysts for the OER at 10 mA cm^−2^ in 1 M KOH.

| Catalyst | Overpotential | Stability | Reference |
| --- | --- | --- | --- |
| Ni_3_Mo-Ni_3_N/NF | 155 mV | 1000 h | This work |
| MgO/NiCo_2_S_4_/CC | 145 mV | 40 h | *Appl. Catal. B Environ.*, **2022,** *312*, 121432 |
| NiCo-PBA/NiFe-LDH | 190 mV | 100 h | *Small*, **2023**, 2309769 |
| MOF-(74+274)/NFF | 198 mV | 120 h | *Adv. Mater.*, **2024**, *36*, 2306910 |
| CoMnLa_0.2_-MOF/CF | 210 mV | 25 h | *ACS Appl. Energy Mater*., **2022**, *5*, 8686-8696 |
| CoS_2_/MS_2_-HPMS | 217 mV | 20 h | *Inorg. Chem. Front.*, **2024**, *11*, 98-106 |
| Fe_2_O_3_/CuO/NF | 220 mV | 100 h | *Adv. Energy Mater*., **2022**, *12*, 2200067 |
| H-Co(OH)_2_/Fe(OOH)_1-x_Cl_x_ | 228 mV | 130 h | *J. Am. Chem. Soc.*, **2023**, *145*, 20261-20272 |
| Co(OH)_2_/NiP_x_/CC | 236 mV | 11 h | *Adv. Funct. Mater.*, **2022**, *32*, 2206407 |
| Ni_0.85_Se-O/CN | 240 mV | 48 h | *Chem. Eng. J.*, **2023**, *454*, 140291 |
| NiOOH/FeOOH/NBs | 246 mV | 120 h | *J. Mater. Chem. A*., **2021**, *9*, 15586-15594 |
| AnBTO200@MOF-Fe/Co | 247 mV | 10 h | *J. Mater. Chem. A.*, **2022**, *10*, 5350-5360 |
| Co/CoMoN/NF | 250 mV | 60 h | *Adv. Sci*., **2022**, *9*, 2105313 |
| NiO/NiS_2_ | 270 mV | 10 h | *Angew. Chem. Int. Ed*., **2022**, *61*, e202207217 |
| CoFe-Co_x_N@NC | 270 mV | 22 h | *AIChE J.*, **2022**, *68*, e17785 |
| WO_3_NRs@Co-MOF/NF | 280 mV | 17 h | *Int. J. Hydrogen Energy*, **2024**, *58*, 1240-1248 |
| RuO_2_/Co_3_O_4_ | 305 mV | 11 h | *RSC Adv.*, **2017**, *7*, 3686-3694 |
| H-CoTe_2_/NiTe_2_@NCBs | 320 mV | 10 h | *Chem. Eng. J.*, **2024**, *486*, 150256 |
| Co_3_ZnC/Co | 366 mV | 0 | *J. Mater. Chem. A*., **2016**, *4*, 9204-9212 |
| FeP/Fe_3_C@NPC | 440 mV | 25 h | *ACS Omega*, **2022**, *7*, 13687-13696 |

Note: NF: Ni foam; CC: carbon cloth; NFF: NiFe foam; CF: carbon fiber; CN: g-C_3_N_4_ nanosheets; NBs: nanoboxes; NC: nanocube; NCBs: N-doped carbon nanoboxes;

**Table S16.** Comparison of recently reported heterojunction catalysts for the overall water splitting in 1 M KOH.

| Catalyst | Potential | Stability | Reference |
| --- | --- | --- | --- |
| Ni_3_Mo-Ni_3_N/NF | η_10_=1.38 V  η_100_=1.54 V | 1000 h | This work |
| Cr-doped FeNi–P/NCN | η_10_=1.50 V | 20 h | *Adv. Mater.*, **2019**, *31*, 1900178 |
| CoFeP-N | η_10_=1.52 V  η_100_=1.64 V | - | *Appl. Catal. B Environ.*, **2024,** *352*, 124027 |
| La_0.5_Sr_0.5_CoO_3-δ_&MoSe_2_ | η_10_=1.52 V | 1000 h | *Nat. Commun.*, **2019**, *10*, 1723 |
| Cu@GDY-Co | η_10_=1.53 V  η_100_=1.70 V | - | *Nano Energy*, **2020**, *74*, 104852 |
| porous MoO_2_ | η_10_=1.53 V | 24 h | *Adv. Mater.*, **2016**, *28*, 3785-3790 |
| Ni_2_P-CoCH/CFP | η_10_=1.53 V | 50 h | *Angew. Chem. Int. Ed.*, **2023**, *62*, e202302795 |
| NiFe–P | η_10_=1.56 V | 12 h | *J. Mater. Chem. A*, **2016**, *4*, 13866-13873 |
| NiFe LDH@NiCoP/NF | η_10_=1.57 V | 100 h | *Adv. Funct. Mater.*, **2018**, *28*, 1706847 |
| La_0.5_Sr_0.5_CoO_3-δ_/K-MoSe_2_ | η_10_=1.59 V  η_100_=1.95 V | - | *Nat. Commun.*, **2021**, *12*, 4606 |
| Ni_2_P | η_10_=1.63 V | 10 h | *Energy Environ. Sci*., **2015**, *8*, 2347-2351 |
| NiCo_2_S_4_ NW/NF | η_10_=1.63 V | 50 h | *Adv. Funct. Mater.*, **2016**, *26*, 4661-4672 |
| Cu_0.5_Fe_0.5_/NF | η_10_=1.64 V | - | *Small*, **2020**, *16*, 1905884 |
| NiS/Ni foam | η_10_=1.64 V | 35 h | *Chem. Commun.*, **2016**, *52*, 1486-1489 |
| CoP/NCNHP | η_10_=1.64 V | 36 h | *J. Am. Chem. Soc.*, **2018**, *140*, 2610-2618 |
| Ni_3_Se_2_/CF | η_10_=1.65 V | 12 h | *Catal. Sci. Technol.*, **2015**, *5*, 4954-4958 |
| (Co_1−x_Ni_x_)(S_1−y_P_y_)_2_/G | η_10_=1.65 V | 50 h | *Adv. Energy Mater*., **2018**, *8*, 1802319 |
| NiCo_2_S_4_ NA/CC | η_10_=1.68 V | 10 h | *Nanoscale*, **2015**, *7*, 15122-15126 |
| NiFeV-LDHs/NF | η_10_=1.59 V | 15 h | *Small*, **2018**, *14*, 1703257 |
| FeCo/FeCoNi-2 | η_10_=1.69 V | 10 h | *ACS Catal.*, **2017**, *7*, 469-479 |

Note: NCN: N-doped carbon nanotube; GDY: graphdiyne; CFP: carbon fiber paper; NF: Ni foam; NW: nanowire; NCNHP: N-doped carbon nanotube hollow polyhedron; CF: carbon fiber; G: graphene; NA: nanowires array; CC: carbon cloth;

**References**

1. G. Kresse, and J. Furthmüller, "Efficient Iterative Schemes for *ab Initio* Total-Energy Calculations Using a Plane-Wave Basis Set," *Physical Review B* 54 (1996): 11169-11186, https://doi.org/10.1103/PhysRevB.54.11169.

2. P. E. Blöchl, "Projector Augmented-Wave Method," *Physical Review B* 50 (1994): 17953-17979, https://doi.org/10.1103/PhysRevB.50.17953.

3. J. P. Perdew, K. Burke, and M. Ernzerhof, "Generalized Gradient Approximation Made Simple," *Physical Review Letters* 78 (1997): 1396, https://doi.org/10.1103/PhysRevLett.77.3865.

4. B. Hammer, L. B. Hansen, and J. K. Nørskov, "Improved Adsorption Energetics Within Density-Functional Theory Using Revised Perdew-Burke-Ernzerhof Functionals," *Physical Review B* 59 (1999): 7413, https://doi.org/10.1103/PhysRevB.59.7413.

5. H. J. Monkhorst, and J. D. Pack, "Special Points for Brillouin-Zone Integrations," *Physical Review B* 13 (1976): 5188-5192, https://doi.org/10.1103/PhysRevB.13.5188.

6. G. Henkelman, and H. Jónsson, "Improved Tangent Estimate in the Nudged Elastic Band Method for Finding Minimum Energy Paths and Saddle Points," *Journal of Chemical Physics* 113 (2000): 9978-9985, https://doi.org/10.1063/1.1323224.

7. J. K. Nørskov, J. Rossmeisl, A. Logadottir, L. Lindqvist, J. R. Kitchin, T. Bligaard, and H. Jo´nsson, "Origin of the Overpotential for Oxygen Reduction at a Fuel-Cell Cathode," *Journal of Physical Chemistry B* 108 (2004): 17886-17892, https://doi.org/10.1021/jp047349j.
